# Role of Interfacial Hydrogen in Ethylene Hydrogenation on Graphite-Supported Ag, Au, and Cu Catalysts

**DOI:** 10.1021/acscatal.4c05246

**Published:** 2024-11-01

**Authors:** Thomas Wicht, Alexander Genest, Lidia E. Chinchilla, Thomas Haunold, Andreas Steiger-Thirsfeld, Michael Stöger-Pollach, José J. Calvino, Günther Rupprechter

**Affiliations:** † Institute of Materials Chemistry, TU Wien, Getreidemarkt 9/BC, 1060 Vienna, Austria; ‡ Departamento de Ciencia de los Materiales e Ingeniería Metalúrgica y Química Inorgánica, Facultad de Ciencias, Universidad de Cádiz, Campus Rio San Pedro, Puerto Real, 11510 Cádiz, Spain; § University Service Centre for Transmission Electron Microscopy, TU Wien, Stadionallee 2/057-02, 1020 Vienna, Austria

**Keywords:** carbon support, ethylene hydrogenation, photoelectron spectroscopy, electron microscopy, density functional theory

## Abstract

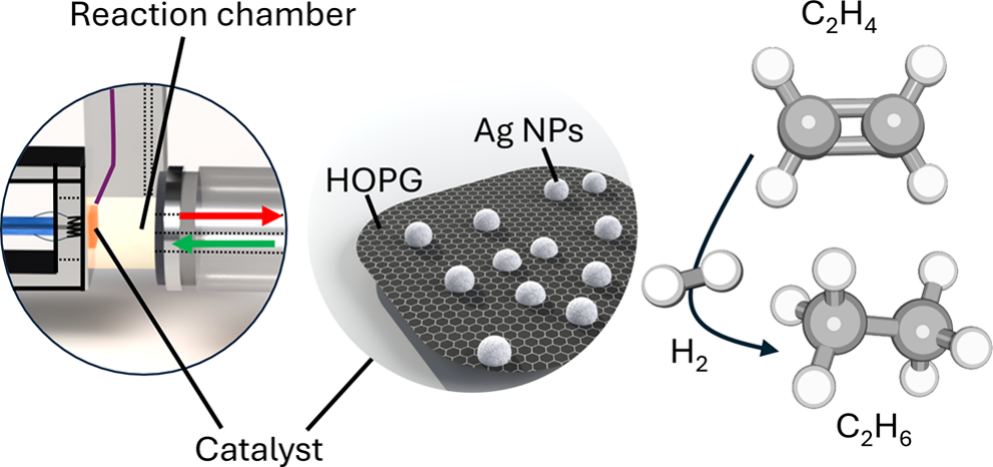

A combined surface science/microreactor approach was applied to examine interface effects in ethylene hydrogenation on carbon-supported Ag, Au, and Cu nanoparticle catalysts. Turnover frequencies (TOFs) were substantially higher for supported catalysts than for (unsupported) polycrystalline metal foils, especially for Ag. Spark ablation of the corresponding metals on highly oriented pyrolytic graphite (HOPG) and carbon-coated grids yielded nanoparticles of around 3 nm size that were well-suited for characterization by X-ray photoelectron spectroscopy (XPS), high-resolution (scanning) transmission electron microscopy (HRTEM/STEM), and energy dispersive X-ray spectroscopy (EDX). Polycrystalline metal foils characterized by scanning electron microscopy (SEM), EDX, electron backscatter diffraction (EBSD), XPS, and low-energy ion scattering (LEIS) served as unsupported references. Employing a UHV-compatible flow microreactor and gas chromatography (GC) allowed us to determine the catalytic performance of the model catalysts in ethylene hydrogenation up to 200 °C under atmospheric pressure. Compared to the pure metal foils, the HOPG-supported metal nanoparticles exhibited not only strongly increased activity but also higher stability (slower deactivation) and differing reaction orders. For the most active Ag catalysts, DFT calculations were carried out to determine the adsorption energies of the reacting species on single-crystal surfaces as well as on carbon-supported and unsupported Ag nanoparticles. Adsorption of molecular hydrogen was very weak on all unsupported Ag surfaces, resulting in hydrocarbon-“poisoned” surfaces. However, when a carbon support was present, the adsorption strength of H_2_ on Ag nanoparticles increased on average by −0.5 eV, driven by changes in Ag–Ag distances near the metal–carbon three-phase boundary (whereas subsurface carbon lowers hydrogen bonding). On Cu particles, the interface effect was calculated to be somewhat weaker than for Ag particles. H_2_/D_2_ scrambling experiments on Ag catalysts then corroborated a facilitated hydrogen activation for carbon-supported metals. Thus, the carbon support effect is attributed to an improved hydrogen availability at the metal–carbon interface, controlling performance.

## Introduction

Carbon is an important support material in heterogeneous catalysis. It exhibits high thermal stability, both in alkaline and acidic media, its chemical properties can be adjusted by varying the surface functional groups, and carbon materials are comparably inexpensive and can be produced from a variety of organic raw materials. Particularly important for catalysis is the fact that carbon supports may exhibit extremely high specific surface areas exceeding 1000 m^2^/g, favoring a high dispersion of active metals, as well as adjustable pore sizes. Recently, it has also become vital to recover the precious metals of spent catalysts, which is especially easy for carbon support materials which can be simply burnt off.^1,2^

Carbon-supported precious metals are widely used in industrial catalysis for fine chemical synthesis and processing, including liquid phase oxidation and hydroprocessing (e.g., hydrogenation, dehydrogenation, hydrodesulfurization), but also for energy generation via fuel cells.^3−6^ Nevertheless, the use of carbon materials, especially “contemporary” ones like carbon nanotubes (CNTs) or templated carbons, is somewhat limited in large-scale industrial processes.^1,6−8^ This may be partly due to lacking knowledge about structure–function relationships, which would allow to optimize catalysts. Apart from the effects of carbon morphology and surface functional groups, metal–carbon phases, subsurface carbon and carbon deposits on the metal may contribute to complex phenomena occurring on a seemingly simple catalyst.^9−13^

Along these lines, ethylene (C_2_H_4_) hydrogenation is a useful well-known benchmark reaction, proceeding via stepwise hydrogenation of the carbon–carbon double bond according to the Horiuti-Polanyi mechanism.^14^ Recently, graphitic carbon-supported Pd and Pt nanoparticles (NPs) have shown superior catalytic performance in this reaction compared to unsupported metal foils, single crystals and oxide-supported NPs (Pd/Al_2_O_3_) of the same metals, manifested by lower activation energies (*E*_a_), lower hydrogen reaction orders, and/or less negative ethylene reaction orders. Higher turnover frequencies (TOFs) of carbon-supported NPs were in common, however.^15−17^ This is particularly surprising as this reaction is generally considered to be structurally insensitive.^18^

The underlying reason for this positive effect of the carbon support has not yet been unraveled. While carbon deposits on metallic catalysts are typically considered to be detrimental to catalyst activity, some reports suggested that carbonaceous deposits may even act as the active phase,^19,20^ as shown, for example, in the dehydrogenation of ethylbenzene over iron oxide catalysts.^21^ Moreover, graphitic supports may affect hydrogenation reactions in ways beyond typical metal–support interactions. Carbon can be used as a hydrogen storage material, pointing to a potential significant role of interfacial hydrogen (hydrogen at the metal–support boundary), hydrogen intercalation, and hydrogen (reverse) spillover in hydrogenation reactions.^22^ Indeed, a beneficial effect of the graphite support on hydrogen availability was suggested,^15,16^ but not yet proven, as hydrogen is difficult to detect.

Regarding the studies on Pd, this is further complicated by the metal’s tendency to form a hydride phase in hydrogen atmosphere. While a general promoting effect of the carbon support was also observed for Pt (which does not form a hydride phase), experimental evidence is lacking as neither reaction orders nor catalyst deactivation were investigated and theoretical studies are lacking. To date, the data available for ethylene hydrogenation on carbon-supported catalysts is scarce, and it is unclear whether similar support effects also occur for other metals. Clearly, the repeatedly reported carbon support effect should be corroborated.

In the work presented herein, Ag, Au and Cu metal NPs were deposited on Highly Oriented Pyrolytic Graphite (HOPG) via spark ablation at atmospheric pressure and room temperature, followed by characterization via X-ray photoelectron spectroscopy (XPS) and (scanning) transmission electron microscopy (TEM/STEM). Their catalytic performance in ethylene hydrogenation was compared to that of (unsupported) reference Ag-, Au-, and Cu-foils, which were examined by scanning electron microscopy (SEM), energy dispersive X-ray spectroscopy (EDX), electron backscatter diffraction (EBSD), XPS and low-energy ion scattering spectroscopy (LEIS). For all catalysts, an atmospheric pressure flow microreactor^23^ and gas chromatographic (GC) gas phase analysis were employed to determine reaction orders, activation energies, turnover frequencies and stability (deactivation) in ethylene (C_2_H_4_) hydrogenation up to 200 °C.

Compared to the pure metal foils, the HOPG-supported metal NPs showed strongly increased activity and stability (slower deactivation), due to better availability of hydrogen at the metal–carbon three-phase boundary. For the most active Ag catalysts, hydrogen–deuterium exchange as well as DFT modeling were carried out to challenge this hypothesis. The H-D exchange was greatly enhanced on the HOPG-supported Ag NPs demonstrating faster hydrogen adsorption and dissociation compared to the unsupported metal foil. Adsorption energies of the reacting species were calculated for unsupported and carbon-supported Ag nanocrystals and single-crystal surfaces. Based on the combined experimental and computational studies, the pronounced support effect could be rationalized by a graphite support-induced increase in hydrogen adsorption energies.

## Results

**Figure 1:**
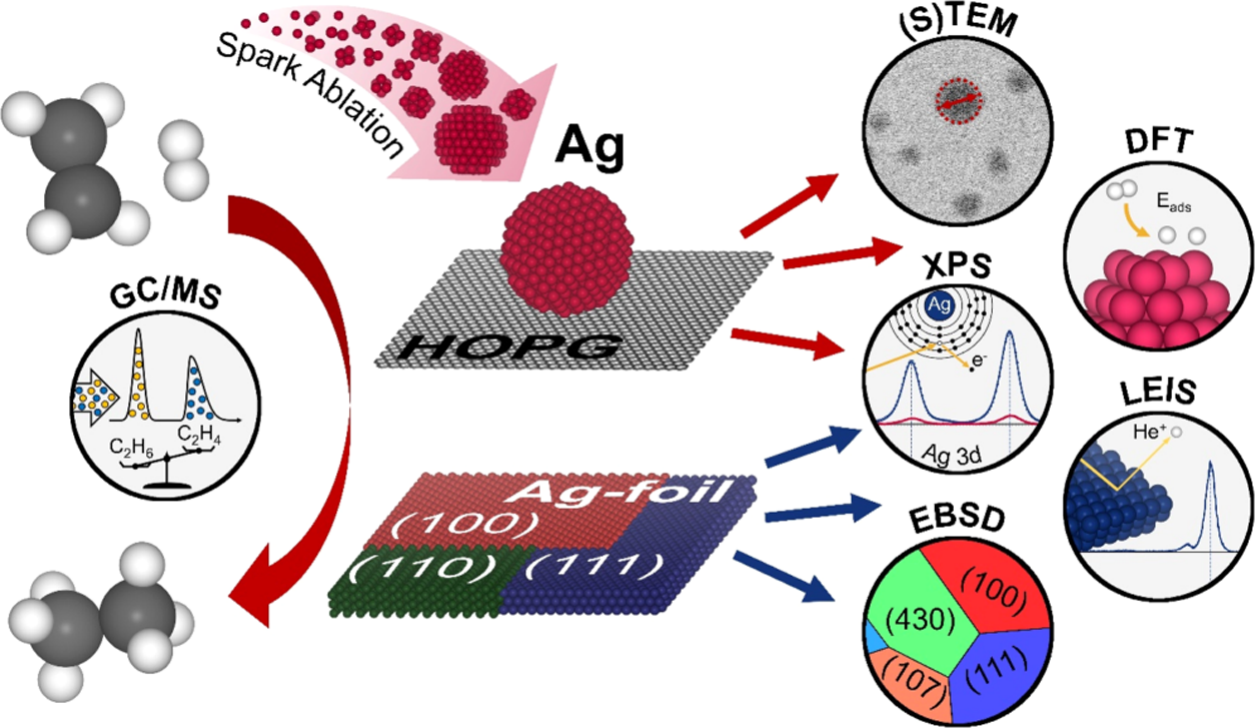
Illustration of the preparation and characterization of the various catalysts tested in ethylene hydrogenation, using Ag as example.

Figure 1 schematically illustrates the preparation, characterization and testing of the various catalysts. Single crystals of HOPG with a size of 7 × 7 x 1 mm^3^ were used as carbon support for the Ag, Au and Cu NPs. The NPs were produced by spark ablation from metal electrodes with a purity of 99.9%, using HOPG and carbon-coated copper TEM grids as substrates. Commercial metal foils of Ag, Au and Cu with a purity >99.9975% were used as references.

Below, the characterization of the various catalysts (after the cleaning procedures described in the Methods section) is presented, followed by kinetic studies and finally DFT modeling.

### Catalyst Characterization

#### Ag Catalysts

**Figure 2:**
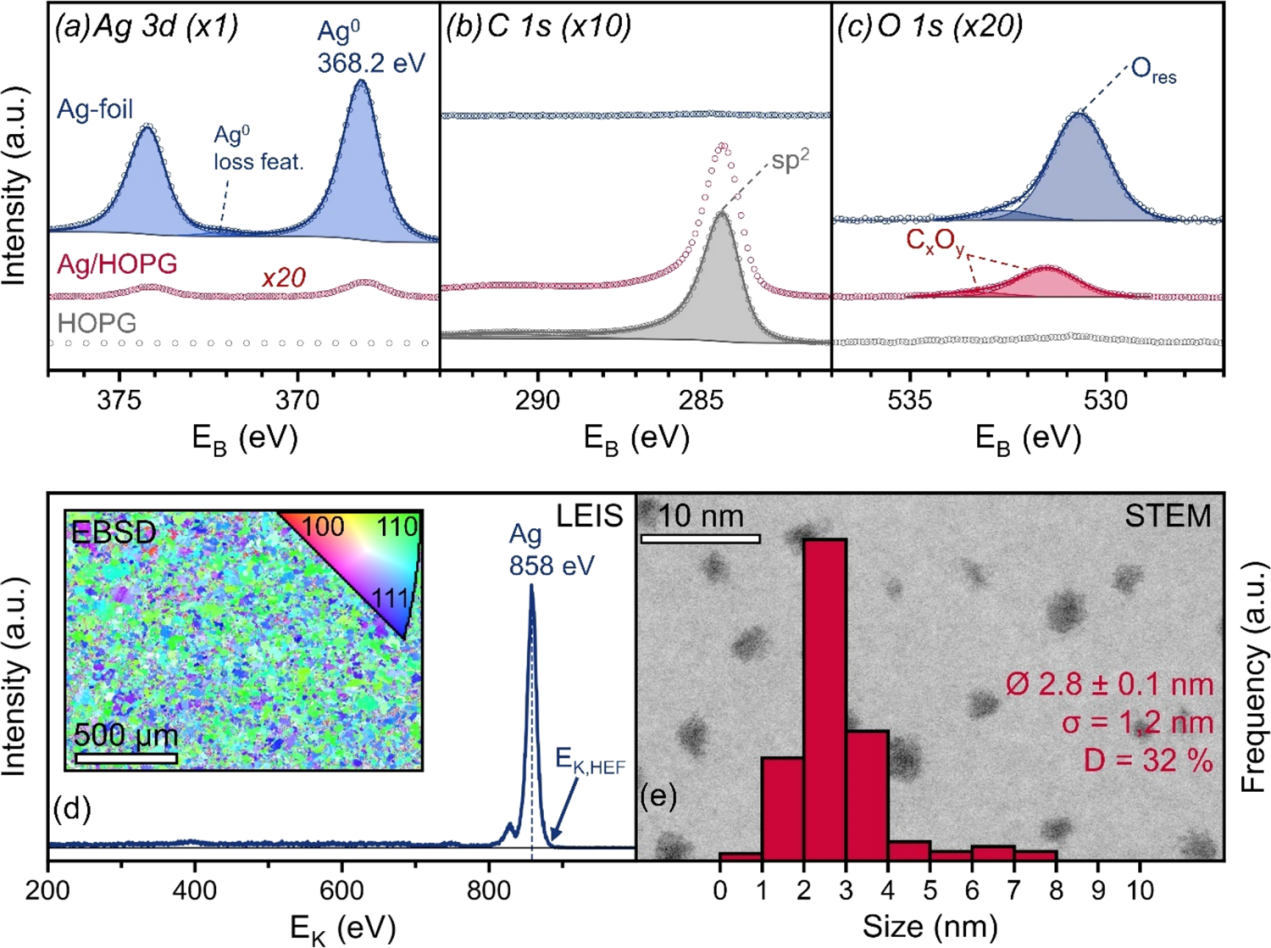
Characterization of Ag-foil, Ag/HOPG and pure HOPG. (a) XPS Ag 3d, (b) C 1s and (c) O 1s spectra of Ag-foil (blue) Ag/HOPG (red) and pure HOPG (gray). (d) LEIS spectrum and EBSD image of Ag-foil. (e) Reversed-contrast HAADF-STEM image of deposited Ag on carbon with the NP size distribution shown as a histogram, and the calculated values for the average particle size Ø, standard deviation σ and the dispersion *D*.

Figure 2a–c displays Ag 3d, C 1s and O 1s XPS spectra, respectively, each comparing Ag-foil, Ag/HOPG and the (pure) HOPG support. For better visibility, some spectra were scaled by a factor indicated in the figure.

For the Ag-foil, the Ag 3d_3/2_ peak position at 368.2 eV,^24^ the presence of the plasmon loss feature, as well as the Auger parameter (α′ = *E*_B_ + *E*_K,Auger_, Figure S2a) of 721.0 eV (Ag^0^:720.7 eV,^24^ Ag_2_O: 719.0 eV,^25^ AgO: 719.5 eV^26^) indicate a metallic oxidation state. The concentration of silver for the foil was 84.6 atom %, showing only traces of adventitious carbon (1.7 atom %) at a binding energy of 284.7 eV, but unexpectedly large amounts of oxygen (13.7 atom %). The O 1s binding energy of 530.7 eV is about 1–2 eV higher than that expected for silver oxides.^25,26^ The origin of this residual oxygen O_res_ might be subsurface O^27−29^ implanted during Ar^+^ sputtering or a contribution from the sample holder (Mo-oxides or steel clips used for sample mounting), as discussed further in Supporting Note 1.

The LEIS measurement of the Ag-foil in Figure 2d shows a very low background, confirming the smoothness of the foil, and a pronounced peak at *E*_K_ of 858 eV, which corresponds to *E*_K,HEF_ (high-energy “foot” of the peak, see methods) of 884 eV, very well in agreement with *E*_K,theo_ of 881 eV for silver. Apart from Ag, only minute amounts of oxygen at 392 eV were visible in LEIS. Next to the Ag main peak another small one appeared at 827 eV (also present for the other metal foils at ∼30–40 eV lower *E*_K_ than the main metal peak), likely a double collision peak (Fe from the sample holder and the steel clips).

EBSD mapping (Figure 2d, inset) of the Ag-foil revealed domains 20–80 μm in size with surface normal orientations mainly along ⟨110⟩, partially along ⟨111⟩, and scarcely along ⟨100⟩ direction.

The HOPG-supported Ag NP sample had a concentration of 0.4 atom % Ag^0^. Given the identical Ag peak position at 368.2 eV the Ag NPs appeared to be metallic as well. The C 1s peak shape is very similar to that of the HOPG reference. In the O 1s spectra, 4.0 atom % of oxygen were detected at 531.5 eV with a small shoulder at 533.3 eV. This reflects a variety of possible oxygen-containing functionalized groups on graphite (i.e., C_*x*_O_*y*_(H_*z*_) compounds at 531–534 eV^30,31^) and/or adsorbed oxygen species from transport through air. For freshly cleaved pure HOPG, the corresponding signals were much smaller.

HAADF-STEM images (Figure 2e) of carbon-supported Ag NPs showed well-separated NPs of 2.8 nm mean size. The corresponding particle size histogram is also included, which shows a size range from 0.5 to 8 nm. This translates to a dispersion of 32% for cuboctahedral shape, which is the lowest energy shape in line with the synthesis of the NPs in gas phase. The contrast of the HAADF-STEM image was inverted for illustration purposes. In (HR)TEM (Figure S13), rounded NPs with fcc structure were observed, so that a cuboctahedral particle shape is a fair assumption.

#### Au Catalysts

**Figure 3:**
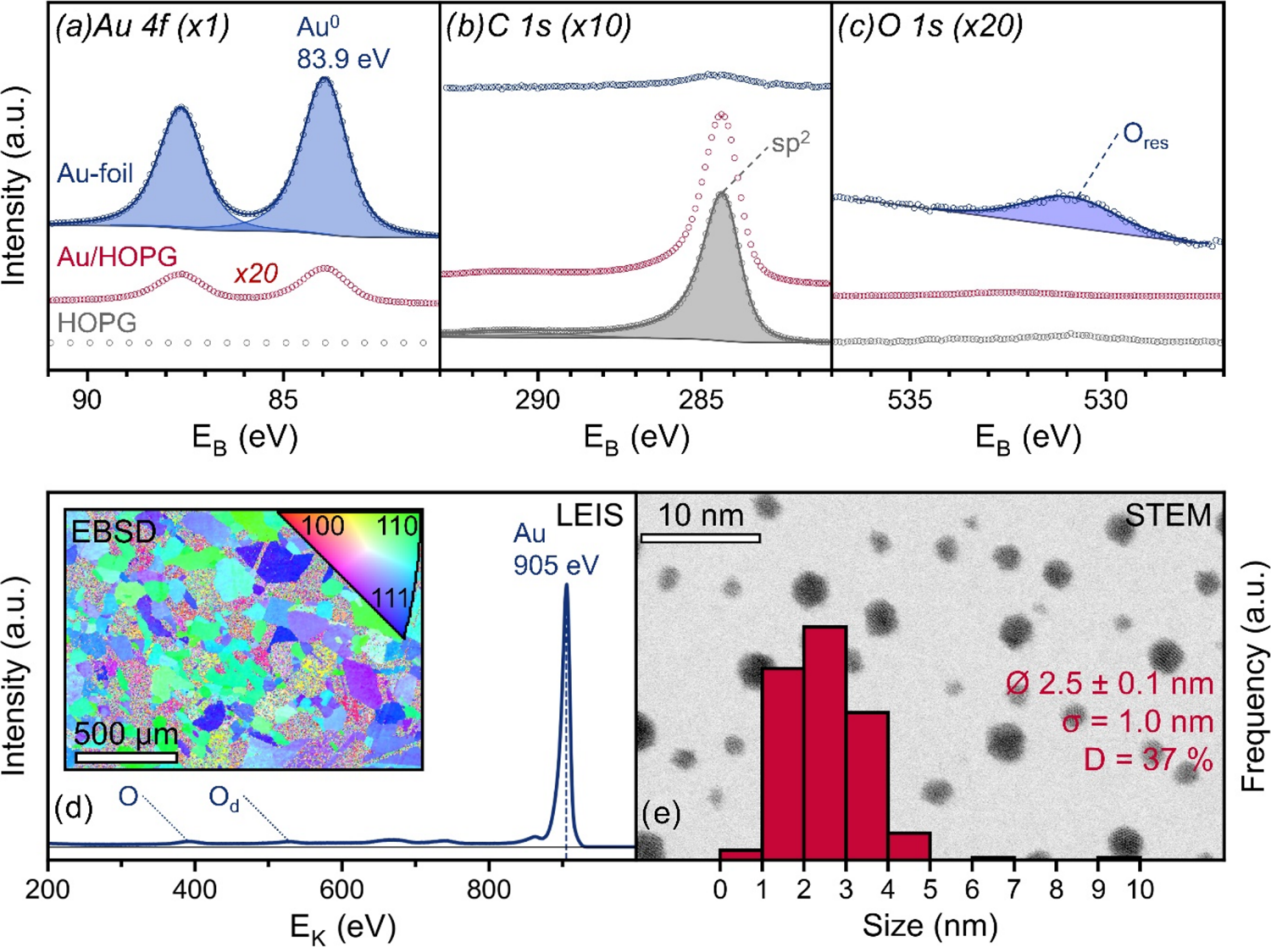
Characterization of Au-foil, Au/HOPG and pure HOPG. (a) XPS Au 4f, (b) C 1s and (c) O 1s spectra of Au-foil (blue) Au/HOPG (red) and pure HOPG (gray). (d) LEIS spectrum and EBSD image of Au-foil. (e) Reversed-contrast HAADF-STEM image of deposited Au on carbon with the NP size distribution shown as a histogram, and the calculated values for the average particle size Ø, standard deviation σ and the dispersion D.

Both the Au-foil and Au/HOPG showed only the Au 4f doublet with the Au 4f_7/2_ peak at 83.9 eV characteristic of Au^0^ (Figure 3a).^32^ The composition of the foil was 80.1 atom % Au, 14.8 atom % carbon and 5.1 atom % oxygen. Again, the oxygen does not seem to stem from gold oxide, as Au_2_O_3_ does not form easily and would exhibit an Au 4f_7/2_ peak ∼2 eV higher and an O 1s peak below 530 eV.^33,34^

The LEIS results confirmed the cleanliness of the foil with a peak of Au at 905 eV (*E*_K_,_HEF_ ≈ 926 eV; *E*_K,theo_ ≈ 933 eV) and a very low background with only tiny amounts of oxygen (O = 392 eV). The EBSD image reveals a mixture of randomly oriented micrograins <5 μm and larger grains of up to 200 μm with preferred surface normal orientations along ⟨111⟩ and ⟨110⟩, with little to no along ⟨100⟩.

In comparison, the Au/HOPG sample had a Au^0^ loading of only 0.4 atom %, 99.0 atom % of sp^2^ carbon and 0.6 atom % of oxygen, the latter likely bound to the graphite or stemming from adsorbates. HAADF-STEM images (Figure 3e) showed Au NPs with about circular projection, a mean size of 2.5 nm and a dispersion of 37% for cuboctahedral shape.

#### Cu Catalysts

**Figure 4:**
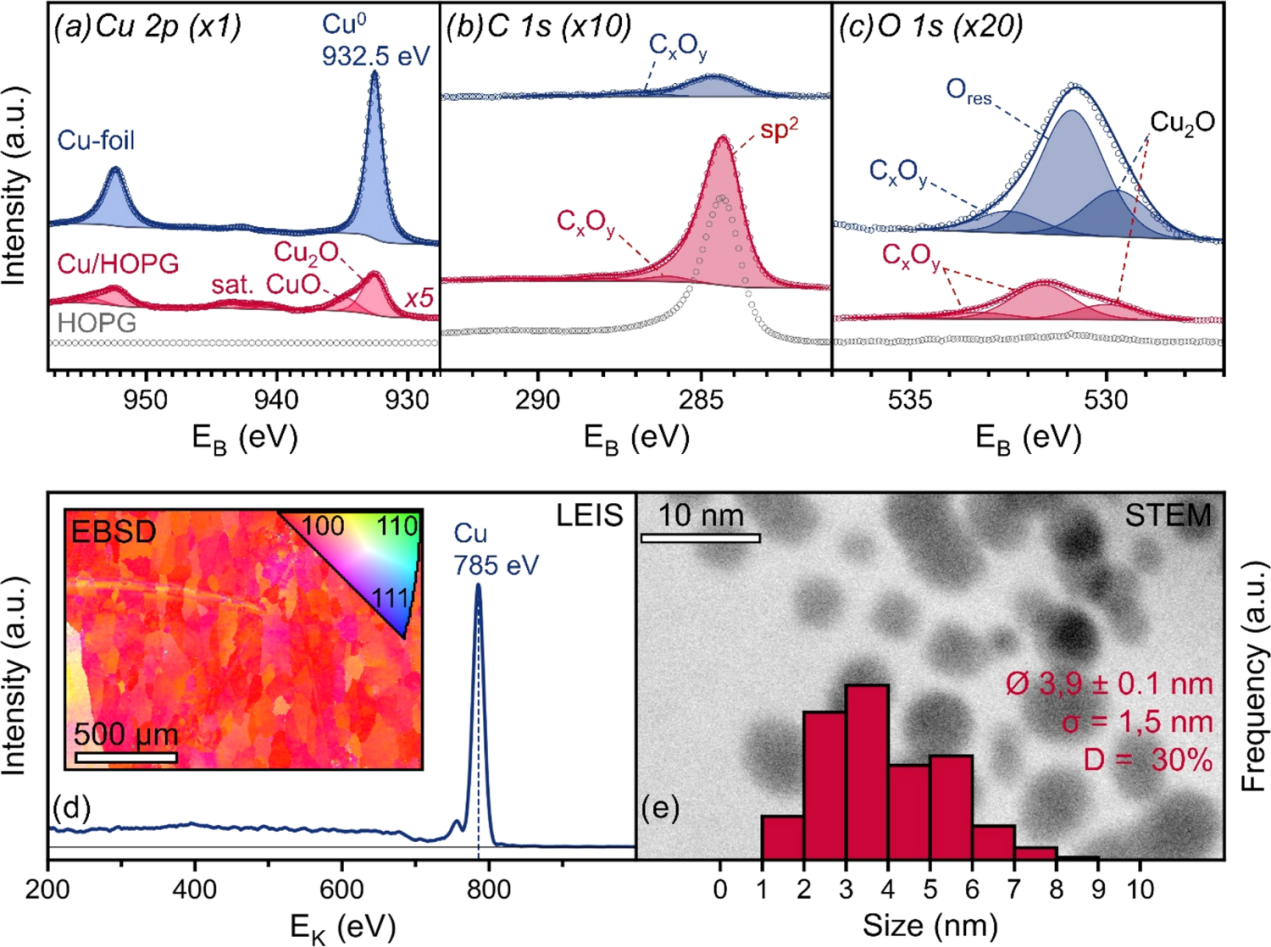
Characterization of Cu-foil, Cu/HOPG and pure HOPG. (a) XPS Cu 2p, (b) C 1s and (c) O 1s spectra of Cu-foil (blue) Cu/HOPG (red) and pure HOPG (gray). (d) LEIS spectrum and EBSD image of Cu-foil. (e) Reversed-contrast HAADF-STEM image of deposited Cu on carbon with the NP size distribution shown as a histogram, and the calculated values for the average particle size Ø, standard deviation σ and the dispersion D.

XPS data of the copper model catalysts are shown in Figure 4a–c. Both have a main Cu 2p_3/2_ peak at 932.6 eV, which can be attributed to either Cu^0^ or Cu_2_O.

For the foil, the α′ (Figure S2b) of 1851.2 eV matches well with literature data for Cu^0^^35^ in contrast to Cu_2_O at 1849.4 eV.^36^ This indicates that the foil consists primarily of Cu^0^. The foil (59.4 atom % Cu) shows 18.1 atom % of carbon as well as 22.6 atom % of oxygen. The O 1s peak can be deconvoluted into 3 different oxygen species. The one around 530 eV most likely corresponds to Cu_2_O,^36^ but the peak area corresponds to only ∼5 atom % Cu_2_O or about 1/10th of the total amount of copper, thus confirming that the Cu-foil is mostly metallic. The second peak is around 531 eV, too high for either Cu_2_O or CuO. Again, it is likely that the peak originates from subsurface O. Lastly, the third peak at 533 eV may correspond to different types of oxidized carbon species C_*x*_O_*y*_.

The LEIS measurement showed a Cu peak at 785 eV (*E*_K_,_HEF_ ≈ 809 eV; *E*_K,theo_ ≈ 806 eV) with high intensity. The EBSD image (Figure 4d) displayed large domains around 100 μm, nearly exclusively oriented along the ⟨100⟩ direction, typical for heavily rolled and tempered Cu.^37,38^

For the Cu/HOPG catalyst, the Cu 2p spectrum displayed the same main peak at 932.6 eV as the foil, but with an additional shoulder at 934.7 eV and satellite peaks (940–945 and 960–965 eV) due to the presence of CuO. The binding energy of the shoulder, which is higher than literature data of bulk CuO at 933.5 eV,^39^ is likely caused by shifts of up to +2 eV reported for small amounts of CuO deposited on support materials. For Cu/HOPG, the α′ value of 1849.6 eV indicates that the main component is Cu_2_O, but Cu^0^ with an α′ shift of −1.7 eV, a realistic value for Cu particles on graphite,^40^ cannot be excluded. The O 1s spectrum shows a total amount of 4.8 atom % related to two peaks of C_*x*_O_*y*_, as well as a third one at 530.0 eV from copper oxides.^39,41^ The amount of O–Cu (∼1.7 atom %) is similar to the total amount of copper deposited (1.5 atom %), suggesting that copper was indeed oxidized after deposition. However, since all samples were reductively pretreated before the kinetic tests, the NPs should be metallic under reaction conditions.^42^ In STEM, the Cu NPs were larger than those observed in the other NP samples (Figure 4e), exhibiting an average size of 3.9 nm and yielding a dispersion of 30% for cuboctahedra.

#### Determining the Number of Exposed Metal Surface Atoms and Summary of Structure Data

The calculation of turnover frequencies (TOFs) requires knowledge of the number of surface metal atoms in a catalyst that are accessible by the reactants. For the metal foils this number of exposed surface atoms was calculated based on the EBSD results. The domains were grouped into the closest matching low-index orientations of either (100), (110) or (110), of which the respective surface atomic densities are known (Figure S3). Considering the distribution of the surface domains, an average surface atomic density was calculated and multiplied with the area of the foil front side, which is accessible to the reactants (0.9 · 7 × 7 mm^2^, ∼10% are covered by steel clips), finally yielding the total number of exposed metal surface atoms for each foil.

For determining the metal surface atoms of the HOPG-supported NPs, a model calculation was applied, combining the XPS and (S)TEM analysis of the catalysts after preparation (as shown in Supporting Note 6, reaction-induced changes are minor). (S)TEM provides the mean size and dispersion (for cuboctahedra) from which an average number of surface atoms per NP can be calculated.

Calculating the number of NPs in a catalyst only from (S)TEM may hold some error, as the number of particles per image is small. Thus, we used the area-averaging XPS results and applied a slightly simplified overlayer model (based on models for specimens of finite and semi-infinite thickness)^43^ to estimate the HOPG surface area that is covered by metal. From that, the total number of NPs and subsequently the total number of exposed metal atoms was determined. (Supporting Note 2)

Table 1 summarizes the structure data of the model catalysts, including the atomic concentrations c_i_ measured by XPS, the mean particle size and dispersion D from (S)TEM, the distribution of the foil surface domain orientation from EBSD, as well as the number of exposed metal surface atoms N_M,S_. In comparison to the NP/HOPG samples, for metal foils, the number of exposed surface metal atoms is about 10 times higher for the Ag and Au samples and about 3 times higher for Cu.

**Table 1:**
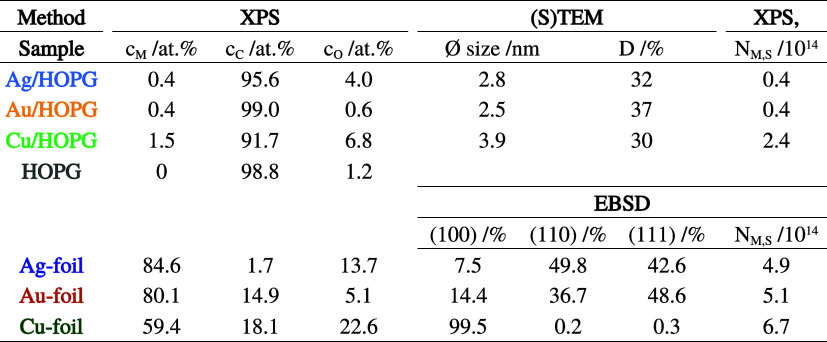
Structure Data of the Various Model Catalysts: XPS Surface Concentrations *c*_i_, (S)TEM Average Particle Size and Dispersion *D*, Distribution of the Foil Surface Domain Orientation from EBSD, and Calculated Number of Exposed Metal Surface Atoms *N*_M,S_

#### Reaction Kinetics

**Figure 5:**
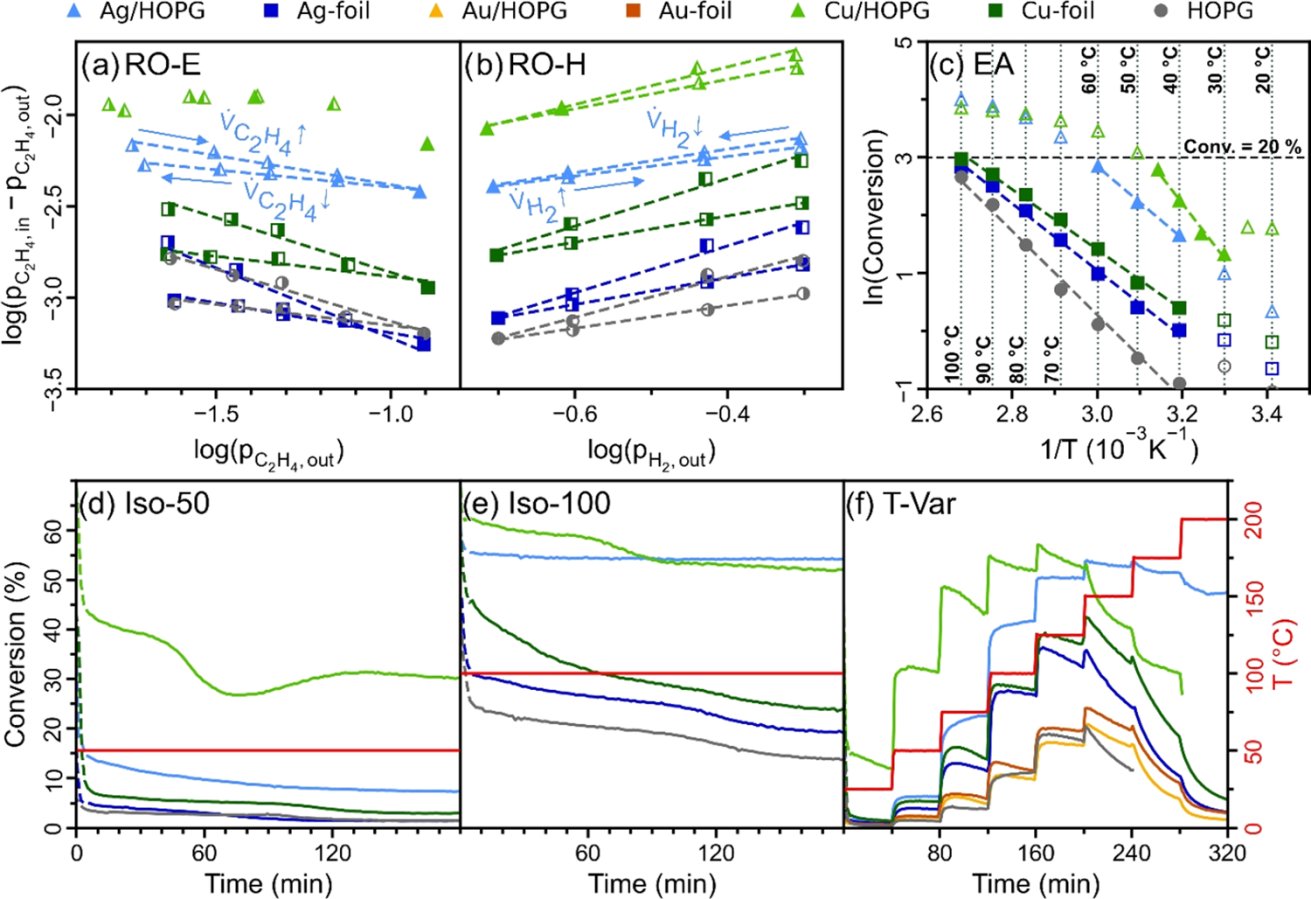
Kinetic experiments comparing the Ag/HOPG, Au/HOPG and Cu/HOPG catalysts, reference Ag-, Au- and Cu-foils, as well as pure HOPG as blind test. The reaction orders of (a) ethylene and (b) hydrogen, (c) the activation energies, the isothermal catalytic performance stability at (d) 50 °C and (e) 100 °C, as well as (f) the overall activity in a broad temperature regime.

Results of kinetic studies of the various Ag, Au, and Cu catalysts are shown in Figure 5 with activation energies (EAs), reaction orders (ROs) and conversions (X) summarized in Tables 2 and 3. In the following sections, the individual experimental runs are discussed. As neither the Au-foil nor Au/HOPG showed significant activity (beyond the blind activity of pure HOPG), they are only shown in Figure 5f for conciseness but will be largely excluded from the discussion.

**Table 2:**
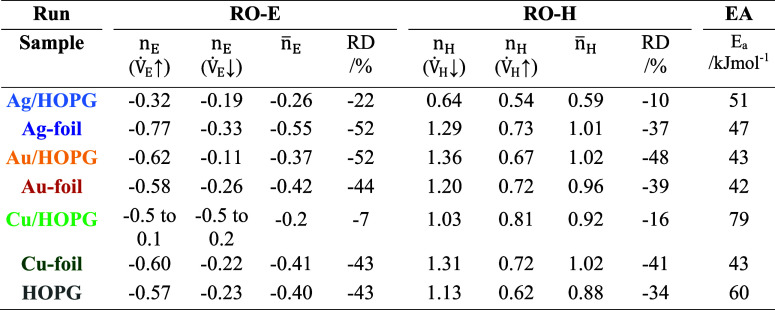
Activation Energies (*E*_a_), Ethylene (*n*_E_), and Hydrogen (*n*_H_) Reaction Orders and Relative Deactivation (RD) Measured for the Different Catalysts in the RO-E, RO-H, and EA Experiments

**Table 3:**
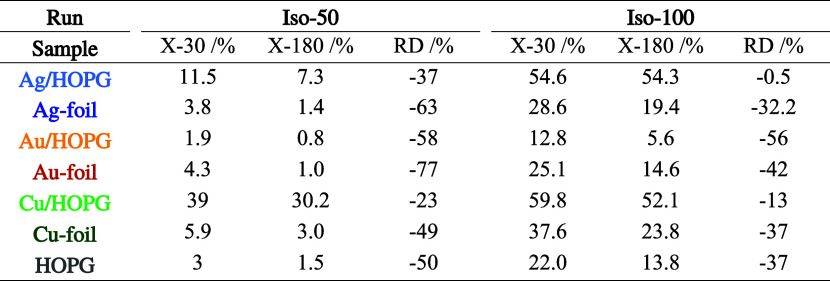
Conversions after 30 min (X-30) toward the Beginning of the Experimental Runs and after 180 min (X-180) at the End of the Experimental Runs, as well as the Relative Deactivation RD Measured for the Different Catalysts in the Iso-50 and Iso-100 Experiments

##### Reaction Order in Ethylene (RO-E)

Reaction rates were measured both for increasing (*V̇*_C_2_H_4__↑) ethylene partial pressures in the first half, and decreasing ethylene (*V̇*_C_2_H_4__↓) partial pressures in the second half of the experiment (other way round for hydrogen), in order to observe and account for catalyst deactivation.

Two general observations can be made: First, the slopes and therefore the ethylene reaction orders (*n*_E_) are negative for all catalysts (except Cu/HOPG at low p_C_2_H_4__) in agreement with values reported for ethylene hydrogenation on other metal surfaces.^18,20,44^

It is well-known, that in ethylene hydrogenation the metal surface is covered to a large extent by hydrocarbon species, possibly blocking the adsorption of hydrogen and thus negatively affecting the catalytic activity.^45−47^ Negative ethylene reaction orders are indicative of this competitive adsorption.^44,48,49^

Surprisingly, the graphite-supported Ag and Cu NPs had less negative ethylene reaction orders compared to unsupported Ag- and Cu-foil. For Cu/HOPG at low ethylene concentrations, there was even a regime in which a slightly positive RO was observed. The deactivation or “poisoning” by ethylene seems to be partially reversible, as can be seen from the reaction rate increasing during the second half of the experiment when the ethylene pressure is lowered again. This regeneration is probably associated with the rehydrogenation and/or desorption of hydrocarbon species, freeing up sites for the adsorption of hydrogen.^50^

This brings us to the second observation, however, as there also seems to be a hysteresis effect due to deactivation that persists when lowering the ethylene pressure, with both the activity and the reaction orders of the second half staying lower compared to the first half of the run. Postreaction (S)TEM and XPS analysis (Supporting Note 6) suggest carbon coking as the main reason for this “irreversible” deactivation (only reversible via oxidative pretreatment).

The difference in reaction orders between the two runs is smallest for Ag/HOPG (only ∼0.13) and Cu/HOPG. Furthermore, the relative deactivation (RD), as determined from the difference in conversion (RD = (X(start)-X(end))/X(start)), is significantly lower for Ag/HOPG (22%) and Cu/HOPG (7%) than for the metal foils (40–50%). Therefore, the HOPG-supported NPs exhibit higher stability against coking and a better tolerance to high ethylene concentrations in general, as evident from the less negative reaction orders.

Catalyst deactivation may, however, also affect the observed reaction orders. As can be seen in Figures 5c, S5 and S6 and as discussed in Supporting Note 3, deactivation mainly impacts the first ∼120 min time on stream after which its effect is rather small. Accordingly, the reaction orders during the second half of the experiment are more reliable.

##### Reaction Order in Hydrogen (RO-H)

The hydrogen reaction orders (n_H_) of the metal foils were typically ∼1.3 in the first and ∼0.7 in the second half of the experiment, indicating strong reaction rate dependence on hydrogen pressure and competitive adsorption with ethylene,^48^ in accordance with literature data for other metal surfaces.^18,20,44^

Once again, Ag/HOPG is an exception, with significantly lower reaction orders of around 0.6 and 0.5, pointing to improved hydrogen availability compared to the pure metal foil. For Cu/HOPG, this effect is less pronounced with a reaction order of ∼1 in the first half, and ∼0.8 in the second half of the run. As mentioned above, due to adsorbed hydrocarbon species hindering hydrogen adsorption, the reaction rate strongly depends on the adsorption and dissociation of hydrogen.^51^

Furthermore, high hydrogen availability may even help in removing these carbonaceous species via rehydrogenation, inhibiting the deactivation of HOPG-supported NPs.^50^ Accordingly, the observed relative deactivation was again lowest for Ag/HOPG (10%), followed by Cu/HOPG (16%), and the other samples with 30–50%.

##### Activation Energies (EA)

The activation energies were calculated from the slopes of an Arrhenius plot. For correct measurements, the concentrations of the reactants in the reactor must be kept approximately constant. Therefore, the activation energy was determined in the low-conversion region of <20%, excluding some data at high temperatures (hollow symbols in Figure 5c). In addition, values were only considered after ∼120 min time on stream, after which only minor deactivation occurred. As Cu/HOPG conversions were >20% already above 50 °C, an additional run was performed with 5 °C temperature steps (up to 50 °C). The data from the two runs was merged. For all resulting interpolation fits, an *R*^2^ > 0.98 was achieved. Most catalysts show activation energies between 40 and 50 kJ/mol similar to literature values for Ni (35 to 50 kJ/mol),^18^ Pt (∼40 kJ/mol),^52^ and Pd (30 to 45 kJ/mol).^53^ Higher activation energies were measured for pure HOPG (60 kJ/mol) and Cu/HOPG (79 kJ/mol).

##### Isothermal Reaction at 50 °C (Iso-50)

At 50 °C, Cu/HOPG yielded by far the highest conversion, starting around 40% and then partially deactivating in a nonlinear manner to ∼30% conversion after 180 min on stream. As for the other experimental runs at 50 °C, the trend of Cu/HOPG > Ag/HOPG > Cu-foil > all others persisted in terms of conversion. All samples showed a high RD of mostly 50–60%, whereby deactivation was more pronounced between 60 and 120 min, and only minor afterward (Figure S5). Cu/HOPG was different, deactivating particularly strongly between 40 and 70 min, but partially regenerating onward and it thus had a RD of only 23%. It is hard to tell what exactly causes this regeneration, as the deactivation by coking is a dynamic process involving a variety of different species and reactions.^54^ Ag/HOPG deactivated rather in an exponential fashion by about 37% which is also less compared to Ag-foil. This, of course, further demonstrates the higher stability of the HOPG-supported catalysts at 50 °C. The conversions at 30 min (X-30, X(start)) and 180 min (X-180, X(end)) served as values for the calculation of the RD.

##### Isothermal Reaction at 100 °C (Iso-100)

At 100 °C, Cu/HOPG and Ag/HOPG had about the same conversion of ∼55%. The difference between these samples was that Cu/HOPG deactivated nonlinearly by around 13%, while Ag/HOPG hardly deactivated at all (0.5%). Following behind in terms of conversion were Cu-foil and Ag-foil, but with much higher RDs of 30–40%. Thus, the HOPG-supported catalysts were also more resistant to deactivation at higher temperatures.

##### Temperature Variation (T-Var)

Consistent with the previous findings, at low temperatures Cu/HOPG had the highest conversions, that were exceeded by Ag/HOPG at 125 °C and above. Cu-foil and Ag-foil also showed significant activity, with the former being more active. The most interesting finding was that Ag/HOPG was the only sample that did not undergo continuous deactivation at high temperatures. Above 125 °C, for all other samples, including Ag-foil and HOPG, conversion decreased drastically with time, more rapidly at higher temperatures, leading to conversions <10% at 200 °C. In contrast, the conversion of Ag/HOPG decreased only slightly at higher temperatures (but not with time), still reaching 47% at 200 °C.

##### Evaluation of the Support Effect

**Figure 6:**
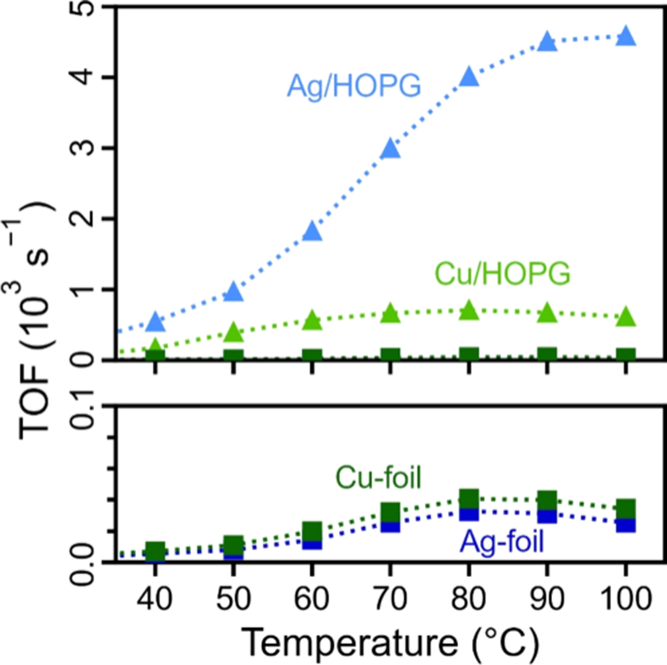
TOFs calculated for the Ag and Cu model catalysts from EA experiments (data acquired after 120 min time on stream, after which deactivation is minor). The blind activity of HOPG was subtracted, which also increases with temperature. For catalysts with low activity, this correction causes a seeming decrease of TOFs at higher temperatures.

For a direct comparison of the supported NP catalysts with the metal foils as well as with literature data, TOFs were calculated. As mentioned, the TOFs are based on the characterization of the as-prepared catalysts since reaction-induced structural changes were minor (Supporting Note 6). Figure 6 displays the TOFs of the EA run from 40–100 °C (only data after 120 min time on stream were considered, after which the effect of deactivation is minor). The TOF values were corrected for “blind activity” by subtracting the conversions of pure HOPG from the conversions of the catalysts (before converting them to TOFs). Additionally, Au catalysts are omitted, as no significant activity was measured. As can be easily seen, the TOFs of the carbon-supported Ag/HOPG and Cu/HOPG exceed by far the ones of their respective (unsupported) metal foils. At 100 °C, Ag/HOPG had the highest TOF of around 4500 s^–1^ followed by Cu/HOPG with 600 s^–1^ and then both Ag- and Cu-foil around 25 and 35 s^–1^ respectively. TOFs of the other runs are shown in Figures S7 and S8, revealing the same trend.

Based on the literature on IB group metal catalysts in ethylene hydrogenation, a rather low activity is expected. Ag and Au are generally considered inactive in alkene hydrogenation.^18,55^ Ag/SiO_2_ exhibited negligible activity compared to Ln(Eu or Yb)-Ag bimetallic catalysts in ethylene hydrogenation.^56^ In another study, oxidatively pretreated Ag/SiO_2_ and Ag/TiO_2_ showed some activity in ethylene hydrogenation, while still being very selective toward the alkene in acetylene hydrogenation.^57^ Cu also provides low activity in ethylene hydrogenation^58^ and is selective in the hydrogenation of butyne and butadiene to butene without formation of the alkane (Cu/SiO_2_ catalyst).^59^

Accordingly, both Ag- (TOF ≈ 8 s^–1^, 50 °C) and Cu-foil (TOF ≈ 11 s^–1^, 50 °C) only show some activity, much lower than that of Pt- (TOF ≈ 120 s^–1^, 50 °C) or Pd-foil (TOF ≈ 220 s^–1^ already at *T* = 20 °C), previously measured in the same microreactor.^23^

Ethylene and, in general, alkene hydrogenation are considered to be structure-insensitive. This means that TOFs should only depend on the number of surface atoms, but not on dispersion or particle size.^18^ Still, we observed strongly increased TOFs for both Ag/HOPG and Cu/HOPG, when compared to Ag- and Cu-foil, respectively. Similar results were observed previously for carbon-supported Pt^15^ and Pd NPs^16,17^ pointing to pronounced support/interface effects.

#### Is It a Structure or a Support Effect?

##### Structure Effect

To contextualize these results, it is worth taking a look at the overall picture. Structural insensitivity in the hydrogenation of alkenes has repeatedly been demonstrated for various catalysts and is generally agreed upon.^20,48,60−62^ Nevertheless, there are some nuances and exceptions, so that the validity of this hypothesis is not entirely beyond question.

For Pd, a particle size effect on ethylene hydrogenation activity was observed by some researchers (1.3–5 nm, atm. pressure),^63^ but not by others (1–3 nm, vacuum).^64^ The same holds true for more complex olefins, where structure sensitivity was reported for cycloalkenes^65^ and allyl alcohol,^66^ while insensitivity^67^ was reported for crotonaldehyde and cis-2-butene-1,4-diol.^68^

In temperature-programmed desorption studies under UHV, Pd thin films or small particles showed higher conversions to ethane than thicker films or single crystals, which exhibit increased hydrogen absorption into the bulk and thus hydrogen depletion at the surface.^69−71^ However, for atmospheric pressure and continuous flow conditions as applied in the current study, this effect should not play a role as the concentration of bulk, surface, and gas phase hydrogen should be in equilibrium.^72,73^ Nevertheless, a further increase in activity has been achieved by adding low-coordinated Pd sites on top of thin Pd films.^74^ Still, as Pd is different from the IB group metals used in our study as it readily forms a stable binary hydride phase (PdH_*x*_) upon exposure to hydrogen gas, it might not be a suitable reference.

For Pt, alkene hydrogenation seems to be both structure and particle size insensitive (except for very small NPs^75,76^).^20,48,77^ Structure insensitivity was also observed for the hydrogenation of linear alkenes on Rh.^78^

Size dependency was observed for Pd/HOPG, with smaller particles (with a higher ratio of interfacial atomic sites) exhibiting higher TOFs, while for Pt/HOPG the effect was rather small. The presence of the carbon support was also decisive regarding activation energies, leading to a reduction of 6–10 kJ/mol.

Thus, structure effects seem incapable of explaining the observed difference between HOPG-supported NPs and the corresponding metal foils.

##### Support Effect

Taking a closer look at the aforementioned studies on graphite-supported catalysts, Pd NPs on HOPG exhibited significantly higher turnover frequencies and lower activation energy (TOF ≈ 3895 s^–1^, *E*_a_ = 29 kJ/mol) than (unsupported) Pd-foil (TOF ≈ 190 s^–1^, *E*_a_ = 34 kJ/mol) at the same reaction conditions (95 °C, p(H_2_) = p(C_2_H_4_) = 72 mbar).^16^ Similarly, HOPG-supported Pt NPs (TOF ≈ 377 s^–1^, *E*_a_ = 25 kJ/mol) exceeded (unsupported) Pt-foil (TOF ≈ 204 s^–1^, *E*_a_ = 36 kJ/mol) under the same conditions (100 °C, p(H_2_) = p(C_2_H_4_) = 72 mbar).^15,16^ Pt/HOPG also outperformed Pt(111) single crystal (TOF ≈ 40 s^–1^, 97 °C, p(H_2_) = 27 mbar, p(C_2_H_4_) = 13 mbar)^20^ or SiO_2_-supported NPs (TOF ≈ 25 s^–1^, 63 °C, p(H_2_) = 150 mbar, p(C_2_H_4_) = 33 mbar).^48^ For temperatures above 40 °C, Pd NPs supported on graphene nanoplatelets (GNPs) showed a higher ethylene reaction order (slightly positive instead of −0.2 to −0.4), a lower hydrogen reaction order (∼0.25), and 3–4 times higher TOFs than very similarly sized Pd particles supported by Al_2_O_3_ or activated carbon (which holds functional groups).^17^

Sarac et al. deposited PdSiAu metallic glass thin films (MGTF) on multilayer graphene (MLGR) supported by Si/SiO_2_, whereby the deposited metals diffused through the graphene lattice so that the MLGR was at the surface. The resulting “sandwich” structure with the MGTF located between Si/SiO_2_ and MLGR exhibited higher hydrogen transfer and electrocatalytic hydrogen evolution activity than the same catalysts without MLGR.^79^

The fact that the metals show higher activity on graphitic than on oxide supports strongly suggests a pronounced support effect. Indeed, a beneficial effect of the graphite support on hydrogen availability was suggested to rationalize these results,^15,16,79^ but evidence is still lacking as hydrogen is difficult to detect experimentally.^80−83^ Once again it is also questionable whether the findings and conclusions from studies on Pd are applicable to other metals that do not easily form a hydride phase. For other graphite-supported metals neither reaction orders nor catalyst deactivation were investigated and in general theoretical studies are lacking. Accordingly, the suggested support effect needs to be corroborated and its origin elucidated.

#### Carbon Support Effect: H–D Exchange and DFT Modeling

Catalysts made of Pd, Pt, Ag, or Cu NPs supported by carbon exhibited much higher turnover frequencies than the respective polycrystalline foils, although the latter also exposed rough surfaces with low-coordination sites.^15−17^ Graphite-supported NPs further exhibited lower reaction orders in hydrogen, less negative reaction orders in ethylene and higher stability against coking.

To verify whether the observed support effects can really be attributed to a higher availability of hydrogen, we performed a hydrogen–deuterium exchange reaction. The same flow microreactor setup was used as for ethylene hydrogenation, with mass spectrometry employed for gas composition analysis. As catalysts, the Ag system was chosen because the TOF-increase induced by the carbon support was highest, which should facilitate the identification of a support effect. As seen in Figure S9, the amount of HD formed is significantly higher (∼5x) for Ag/HOPG than for Ag-foil, despite the lower metal loading. This confirms that the rate of hydrogen adsorption and dissociation is enhanced for the graphite-supported catalysts.

To rationalize the effect of the carbon support, we further carried out DFT calculations, contrasting carbon-supported Ag NP models with Ag single-crystal surfaces of (111) and (100) orientation, as well as with unsupported NPs.

To model Ag/HOPG, a Ag_37_ NP supported by a graphene layer was used. The Ag NP is ∼1 nm in diameter, somewhat smaller than the NPs in the experiment, but with its 3 layers in height all atoms should still be affected by the interface, according to ref.^84^ In the NP, 19 Ag atoms interact directly with the carbon support, 12 Ag atoms are in the middle layer, 6 atoms at the top facet. Thus, Ag_37_ has 27 surface atoms, with the remaining 10 Ag atoms being inside (70% dispersion). This NP model has (111) top and bottom facets and alternating (100) and (111) side facets.

To screen various adsorption sites at reasonable computational times, HOPG was represented by a fully optimized defect-free single graphite (graphene) layer. If anything, this may only underestimate an attractive interaction between the Ag_37_ or its reactants and the carbon support. The optimization of adsorbates was started at 1.5 Å distance from all considered surface atoms.

**Figure 7:**
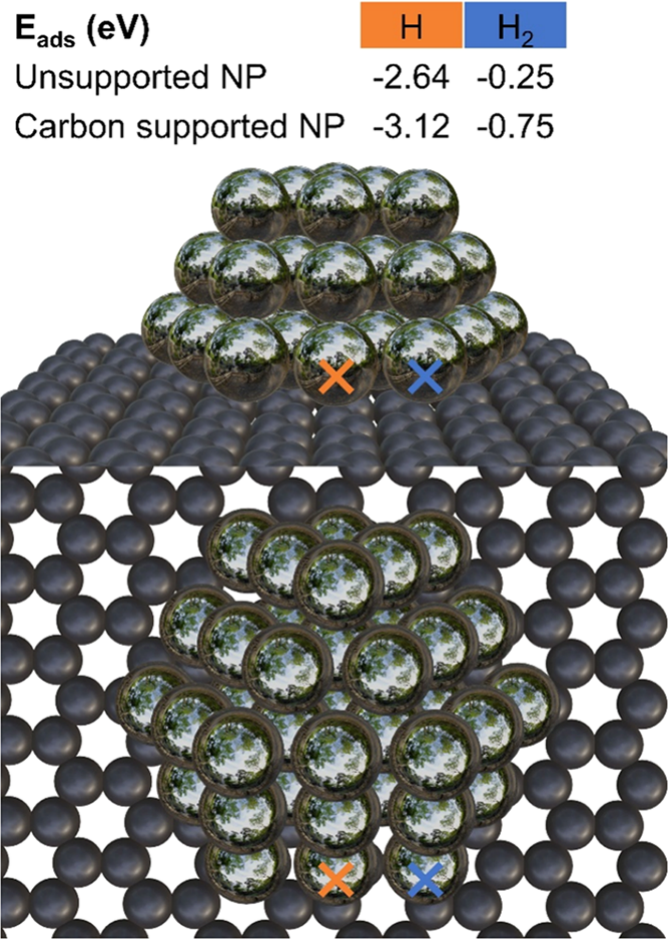
Graphene-supported Ag_37_ nanoparticle model. The adsorption energies of atomic and molecular hydrogen are significantly higher on the carbon-supported Ag NP than on the unsupported NP.

Molecular H_2_ is known to bind weakly at silver single-crystal surfaces,^85^ which is also reflected in the present adsorption energy calculations with *E*_ads_(H_2_) = −0.07 eV (Supporting Note 5). Adsorbing H_2_ on an unsupported Ag NP is already more preferred with *E*_ads_(H_2_) = −0.25 eV, but the highest binding energy was determined for a graphene-supported Ag NP, with *E*_ads_(H_2_) = −0.75 eV at the edge directly at the interface (blue cross in Figure 7).

Although a single H atom binds much stronger, the trend is quite analogous: ∼2.0 eV on single-crystal surfaces, between 2.0 and 2.5 eV at a bare Ag NP, and strongest at a graphene-supported Ag NP with 2.5 to 3.1 eV (orange cross in Figure 7). Interestingly, the smallest adsorption energies are determined at the edge sites, largest energies at the bottom perimeter, independent of the facet (Figure 7 and Supporting Note 5).

Thus, in the absence of the graphene support, H_2_ adsorption is insufficient, because the typical entropy loss of adsorption processes, 0.20 eV calculated for 300 K,^86^ must be overcome. This leads to a lack of hydrogen supply at the pure Ag surface due to its low H_2_ adsorption energies. Vice versa, this highlights the importance of the carbon support in making H_2_ adsorption favorable. Other adsorbates were also studied, as presented in the Supporting Information (Supporting Note 5).

Previously, carbon support effects on hydrogenation have been traced back to variations in the local charge of Pd atoms within the first 3 layers of a NP.^9,84^ In the current work, various possible effects of the carbon support were inspected: a detailed analysis confirmed essentially no influence of the Mulliken charge at the Ag in question, changes remained below 0.01 eV even in the bottom layer of the Ag_37_ NP, which is expected for defect-free graphene. Still, the binding energy of the Ag NP to the graphene layer of 3.6 eV points to a pronounced interaction.

Furthermore, when the graphene support is present, the metal–metal distance in the bottom layer of the NP is expanded by up to 0.03 Å. Thus, the graphene sheet may alter the lattice of the directly adjacent Ag layer, which in turn leads to higher binding energies in light of a bond competition model, which assigns higher bond strengths to (slightly) lower coordinated atoms.

Alternatively, carbon could have been incorporated into the Ag nanoparticle, which may alter its catalytic properties. For example, in Pd NPs, the presence of carbon below the metal surface improves the catalytic performance in olefin hydrogenation,^87^ which was rationalized by DFT modeling showing a reduced binding energy of atomic H on the surface which thus facilitated H migration into the subsurface.^88^ We selected a location of carbon in Ag, close to the graphene layer and studied its effect of the adsorption of H_2_ vs H, (Figure S11 and Tables S4 and S5). Interestingly, H adsorption is only somewhat affected, reducing its binding strength by 0.1 to 0.6 eV, approximately compensating for the effect of the graphene support. H_2_ adsorption at Ag with subsurface carbon is comparable to that at the bare Ag particle, with an adsorption energy between −0.2 and −0.1 eV, giving rise to desorption before activation, as discussed for the bare particle. Therefore, we expect subsurface carbon not being a decisive factor in our case. Some exploratory calculations on the most promising sites for Cu revealed a similar but weaker effect (Table S4). Furthermore, Ag, Cu and Au do not form stable binary hydrides in hydrogen atmosphere under typical reaction conditions, so that the presence of subsurface hydrogen is highly unlikely.^89^

Consequently, a carbon support like graphite has a strongly favorable effect on H_2_ adsorption and a moderate one on the general potential energy surface. Although differences between the sites of the NP are small, the overall promoting effect of carbon supports is evident. Furthermore, the favorable hydrogen adsorption on carbon-supported NPs may inhibit or slow down deactivation by coking. A related case was reported for CO oxidation, when the perimeter sites of oxide-supported Pd particles bind oxygen more strongly (as compared to unsupported Pd particles) and thereby exhibit an increased resistance against CO poisoning.^90^

## Conclusions

Ag, Au, and Cu nanoparticle catalysts were manufactured via spark ablation on HOPG and carbon-coated TEM grids, producing well-defined model catalysts (mean particle size ∼3 nm) suitable for characterization by XPS and (S)TEM/EDX. Polycrystalline metal foils, thoroughly analyzed by XPS, LEIS and SEM/EBSD, served as reference catalysts. The catalytic performance of the various catalysts in ethylene hydrogenation was quantitively determined at atmospheric pressure using a UHV-compatible flow microreactor with GC analysis.

Ethylene hydrogenation is considered structure-insensitive both in terms of particle size (apart from tiny NPs or clusters^75,76^) and surface orientation.^48,60−62^ Herein, we have focused on the support effect by comparing HOPG-supported NPs with unsupported metal foils, revealing strongly increased TOFs for both Ag/HOPG and Cu/HOPG, when compared to unsupported Ag- and Cu-foil, respectively (Au catalysts had only minute activity).

Furthermore, the HOPG-supported NPs showed lower hydrogen reaction orders, less negative ethylene reaction orders, and improved stability against deactivation by coking. Similar promotion effects by a carbon support had been reported by some of us for Pd and Pt NPs. Altogether, this clearly points to an improved hydrogen availability for carbon-supported catalysts. This was further substantiated by increased H-D exchange on the graphite-supported catalyst, and rationalized by DFT modeling, which showed that graphene has a favorable effect on hydrogen adsorption by inducing structural changes in the metal NP, resulting in stronger hydrogen bonding especially at the perimeter sites. This is crucial, as both the rate of ethylene hydrogenation and of rehydrogenation of coke species depend critically on the amount of hydrogen adsorbed on the metal catalyst.

The effect of a carbon support thus extends beyond promoting high dispersion, as evident from the present activity data normalized to the available surface metal atoms. The ability to enhance catalyst performance through tuning hydrogen adsorption via the choice of a suitable support holds significant promise for the further design and optimization of hydrogenation catalysts.

## Methods

### Catalyst Preparation and Characterization

Figure 1 schematically illustrates the preparation, characterization and testing of the various catalysts. Single crystals of HOPG (ZYA grade, mosaic spread 0.4–0.7°, NT-MDT) with a size of 7 × 7 x 1 mm^3^ were used as carbon support for the Ag, Au and Cu NPs. To prepare a smooth and clean surface, the topmost layers of the HOPG crystals were peeled off with sticky tape.

The metal NPs were produced by spark ablation from 3 mm thick electrodes of Ag, Au and Cu with a purity of 99.9% using a commercial VSP-G1 nanoparticle generator (VSPARTICLE, Delft, NL) operated at 1 kV, 5–8 mA and using Ar as a carrier gas. Using the VSP-A1 diffusion accessory, particles form in the gas phase at room temperature (RT) and atmospheric pressure (AP). Deposition is based on diffusion, as the HOPG substrates were mounted parallel downstream of the flow. Additionally, carbon-coated copper TEM grids were mounted next to the HOPG crystals, so that analogous NPs were deposited on both types of substrate. A similar spark ablation approach has been reported for Ni NPs on Au@SiO_2_ wafers^91^ and for Ni NPs onto the SiN membrane of an in situ TEM MEMS reactor,^92^ and HOPG also served as substrate for PVD of Pt^15^ and Pd NPs.^16,93^

Commercial metal foils (Alfa Aesar) of Ag, Au and Cu with a purity >99.9975% and a thickness of 0.1 mm were used as references to determine the activities of unsupported metals. Square pieces of 7 × 7 mm^2^ size were cut, polished and cleaned with isopropanol in an ultrasonic bath for 15 min.

Both the NP/HOPG catalysts and the metal foils, mounted to Mo or Ta sample holders by steel clips, were then transferred to a UHV system.^23^ The foils were further cleaned inside the UHV chamber by repeated cycles of oxidation (5 × 10^–6^ mbar O_2_, 400 °C, 10 min), Ar^+^ sputtering (5 × 10^–6^ mbar Ar, 1.5 keV, RT, 10 min), reduction (5 × 10^–6^ mbar H_2_, 400 °C, 10 min) and annealing (UHV, 500 °C, 10 min) between each step until clean (no carbon visible in XPS survey spectra). The NP/HOPG catalysts were characterized in the as-prepared state (after spark ablation synthesis).

### HRTEM, STEM, and SEM/EBSD

The NPs deposited on carbon-coated TEM grids were first analyzed in the as-prepared state, but also a second time after pretreatment and exposure to reaction conditions (two preparation cycles, same conditions as described below in Reaction Kinetics). Different transmission electron microscopy (TEM) techniques were used to characterize all samples, including high-resolution TEM (HRTEM), scanning transmission electron microscopy (STEM) collecting scattered electrons in a high-angle annular dark-field (HAADF) detector, X-ray energy dispersive spectroscopy (EDX), and selected area electron diffraction (SAED). Altogether, these techniques enabled to study the size distribution, morphology, and composition of the synthesized NPs.

The size distribution of the particles was directly retrieved from HRTEM and HAADF-STEM images by analyzing the size of about 300 NPs for each sample. The total metal dispersion (*D*) was calculated from all particles counted according to a cuboctahedra particle model, in which *D* = ∑*N*_S_/*N*_T_, where *N*_S_ represents the number of surface atoms in a metal particle and *N*_T_ is the number of atoms in a metal particle.^94^

The microscope used was a FEI Titan^3^ 60–300 equipped with a high coherence and brightness field electron gun (X-FEG) and fitted with spherical aberration correctors on the probe and image-forming lens systems. It was operated at an accelerating voltage of 200 kV for the characterization of Au and Ag samples, and 80 kV for the Cu samples, which were found sensitive to the high-intensity electron beam.

The metal foils were characterized by scanning electron microscopy (SEM, Figure S1), EDX (Table S1), and electron backscatter diffraction (EBSD) in a field emission scanning electron microscope (FEI Quanta 200F) using standard EBSD conditions and evaluation procedures^95^ to determine the dimension and the crystallographic orientations of the (hkl) domains present. For EBSD analysis, backscattered electrons of an electron beam, focused on a particular region of a sample, form a backscatter Kikuchi diffraction pattern, which corresponds to each of the diffracting crystal lattice planes.^96,97^

### XPS and LEIS

All catalysts were analyzed by X-ray photoelectron spectroscopy (XPS; Specs XR50© high-intensity nonmonochromatic Al/Mg dual anode X-ray source and Phoibos 100 energy analyzer (EA) with multichannel plate). Spectra were obtained at RT, at an emission angle of 0° and using an Al anode with K_α_ radiation at 1486.6 eV. Data analysis was performed via CasaXPS. For NP/HOPG, the binding energy in the spectra was referenced to the C 1s peak at 284.3 eV,^98^ whereas for metal foils calibration was based on their Fermi edge. The spot size of the X-ray beam is about 10 mm in diameter, so that accompanying signals of steel clips holding the sample may occur for small samples.

For the metal foils, the surface composition and cleanliness were further analyzed by low-energy ion scattering (Specs IQE 12/38© ion source, detection via EA above) using He^+^-ions at a kinetic energy of 1 keV and a scattering angle of 135°. The employed setup and method have been described in detail elsewhere.^99^ The peak identification was supported by theoretical calculations of a material’s specific kinetic energy *E*_K,theo_, which corresponds to the high-energy “foot” (HEF) of the peak.^100^ For comparison, this value is read out from the experimental data as the kinetic energy *E*_K_,_HEF_ at which the corresponding intensity reaches ∼1% of the maximum peak intensity.

### Reaction Kinetics

For kinetic tests, the catalysts were transferred into a UHV-compatible microreactor setup, described in detail previously.^23^ The samples were mounted in a 4 mL stainless-steel reaction cell with stainless-steel clips. A Ta filament located outside the reaction cell was used to heat the backside of the wall where the catalyst was mounted. Since the wall thickness next to the filament is only 1 mm and the reaction cell additionally water-cooled, the catalyst can be effectively heated, while the temperature of the other cell walls stays low. The catalyst temperature was measured inside the reactor by a Ni/Ni–Cr thermocouple adjacent to the catalyst.

The reactor was operated under continuous flow conditions at atmospheric pressure. The feed was controlled by four mass flow controllers, regulating the volumetric gas flows *V̇* of Ar, H_2_, O_2_ and ethylene (C_2_H_4_). Product analysis and quantification were done by gas chromatography (GC; Micro GC Fusion, INFICON) and mass spectrometry (MS; Hiden HPR 20©).

Before running kinetic tests, all samples were pretreated by cycles (1h each) of oxidation (10 vol % O_2_ in Ar ≙ 100 mbar O_2_, *V̇*_total_ = 15 mL/min, 150 °C) to remove potential carbon contamination, reduction (10 vol % H_2_ in Ar ≙ 100 mbar H_2_, *V̇*_total_ = 15 mL/min, 150 °C) to re-establish metallic oxidation states, and exposure to reactants (*V̇*_H_2__ = 5 mL/min, *V̇*_C_2_H_4__ = 1 mL/min, *V̇*_Ar_ = 14 mL/min, 100 °C) until initial reactivity was constant. Before every consecutive reaction run, catalysts were again pretreated (oxidation and reduction, same as above).

All reaction runs were carried out at an inert gas flow rate of *V̇*_Ar_ = 14 mL/min and a total flow rate of 20 mL/min. Unless otherwise specified (such as for reaction order measurements for ethylene (RO-E) or hydrogen (RO-H)), the reactant flow rates were *V̇*_H_2__ = 5 mL/min and *V̇*_C_2_H_4__ = 1 mL/min (the latter corresponding to 0.045 mmol/min or 4.5 × 10^17^ ethylene molecules per second).

The following experimental runs were carried out:

The flow (partial pressure) of ethylene was varied in order to determine its reaction order. It is worth noting that an ethylene-rich atmosphere (high C_2_H_4_/H_2_ ratio) can lead to catalyst deactivation, which may or may not be reversible once the ethylene concentration is reduced again. Knowing which type of deactivation is present is not only crucial for industrial applications of the catalysts, it is also important when determining the ethylene reaction order. Irreversible deactivation would lead to a lower observed reaction order when increasing the ethylene concentration compared to when decreasing it. In order to be able to both observe and account for this effect, the initial ethylene flow rate was chosen as low as 0.5 mL/min, increased in steps every 40 min to 2.5 mL/min and then decreased again to 0.5 mL/min. A comparison of the reaction orders and reaction rates of the “two halves” of the experiment (increase and decrease of ethylene flow) then allows to determine the type and extent of deactivation. As the conversion was kept low via the low reaction temperature and hydrogen added in excess, the hydrogen concentration in the reactor was virtually constant.

The reaction order in ethylene (*n*_E_) was determined from the slope of a double logarithmic plot of the difference in ethylene partial pressure (in bar) between reactor inlet (p_C_2_H_4_,in_) and outlet (p_C_2_H_4_,out_) vs the ethylene partial pressure at the catalyst (∼p_C_2_H_4_,out_). The data points were averaged using the last 5 min of the 40 min intervals of constant reaction conditions.
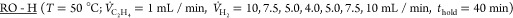


The flow (partial pressure) of hydrogen was varied in order to determine its reaction order. For RO-H, the initial hydrogen flow rate was chosen as high as 10 mL/min, decreased in stepwise fashion every 40 min to 4 mL/min and then increased again to 10 mL/min (Figure S6). As the conversion was kept low via the low reaction temperature, the ethylene concentration in the reactor remained nearly constant.
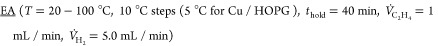


Conversion was measured for temperatures increasing from 20 to 100 °C in order to calculate the activation energy.
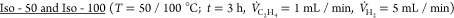


All reaction parameters were kept constant at 50/100 °C to study whether catalytic performance was stable at lower/higher temperature.
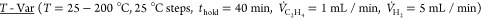


Conversion was measured for increasing temperatures to compare catalyst activities over a wide temperature regime.

With the given setup and the chosen reaction conditions, some well-founded assumptions simplify the calculations: First of all, no side reactions (except for some coking) take place apart from ethylene hydrogenation, as no byproducts were detected by GC or MS. Furthermore, the volumetric flow rate at the inlet and outlet of the reactor was constant and basically equal, as ethylene is converted equimolar to ethane, and ample inert gas as well as excess hydrogen were present. Accordingly, the retention time remained constant. Lastly, the reaction only took place inside the reactor and mostly at the heated catalyst mounted to the backwall, assuming the reactor is ideally mixed. Therefore, the gas composition measured by GC corresponds to the one inside the reactor and at the catalyst.

### Hydrogen–Deuterium Exchange Reaction

For the H-D exchange reaction the same microreactor setup as described above was used, with online gas phase composition analysis performed using a quadrupole mass spectrometer (MS, Omnistar, Pfeiffer Vacuum, SEM detector). The samples were pretreated by oxidation and reduction (same conditions as above, 1h each). Additional information can be found in Supporting Note 4.

### DFT Modeling

The DFT calculations were carried out with the plane-wave based program package VASP^101,102^ using the PBE^103^ functional and the PAW method.^104,105^ An energy cutoff of 450 eV was used throughout. Nanoparticle models were computed using a 1 × 1 × 1 grid, the low-index surfaces by a 3 × 3 × 1 Monkhorst–Pack grid,^106^ both types with first-order Methfessel-Paxton smearing (sigma = 0.05 eV).^107^ Van-der Waals interactions were included using the D3 method integrated in VASP.^108^

The supported catalyst model consisted of a 3-layer Ag_37_ NP, exposing a (111) top facet and (111) and (100) side facets, and a defect-free single-layer graphite (graphene) sheet of 160 carbon atoms. Support effects have previously been shown to affect solely the bottom 3-layers of a supported metal,^84^ so that Ag_37_ is sufficient to mimic the experimentally studied ∼3 nm NPs.

The single-crystal models of (111) and (100) type were modeled by 4 × 4 Ag atoms per layer, with three layers in total and the “bottom” two fixed at their calculated bulk positions, (Ag–Ag distance 289 pm). A vacuum spacing of 1 nm between the repeated slabs was adopted.

To rationalize the differences between supported and unsupported nanoparticles, a specific procedure was followed. Once a supported nanoparticle with or without adsorbate was converged, the carbon support was removed from this structure, before the remaining Ag_37_ moiety with or without adsorbate was used as a starting structure for subsequent relaxation. This should ensure that a difference in adsorption energy between supported and unsupported nanoparticles is truly related to a support effect.

In all cases, adsorption energies were calculated as *E*_ads_(*x*) = *E*(*x*@Ag_37_) – *E*(*x*) – *E*(Ag_37_), where *x* denotes the adsorbate in the gas phase, Ag_37_ is the pure metal NP and x@Ag_37_ a structure with the adsorbate bound to the NP. With this definition, a negative value of *E*_ads_ implies a favorable adsorption process.

## Supporting Information Available

SEM, EDX, XPS Auger regions and O 1s region details, surface site calculations, additional kinetic data, H-D exchange, DFT modeling details and full results, postreaction analysis of NPs by (HR-)TEM and (S)TEM, deactivation studies (PDF)
