# Supplementary material for: Role of Interfacial Hydrogen in Ethylene Hydrogenation on Graphite-Supported Ag, Au, and Cu Catalysts
Source: ACS Catal. 2024 Nov 1;14(22):16905–19. doi: 10.1021/acscatal.4c05246 (PMC11574765; doi:10.1021/acscatal.4c05246)
Supplement: Supplementary file 1 — cs4c05246_si_001.pdf [file cs4c05246_si_001.pdf]

Supporting Information

The role of interfacial hydrogen in ethylene hydrogenation on graphite-supported Ag, Au, and Cu catalysts

*Thomas Wicht[1], Alexander Genest[1], Lidia E. Chinchilla[2], Thomas Haunold[1], Andreas Steiger-Thirsfeld[3], Michael Stöger-Pollach[3], Jose J. Calvino[2], Günther Rupprechter[1]**

[1]*Institute of Materials Chemistry, TU Wien, Getreidemarkt 9/BC, 1060 Vienna, Austria*

[2]*Departamento de Ciencia de los Materiales e Ingeniería Metalúrgica y Química Inorgánica, Facultad de Ciencias, Universidad de Cádiz, Campus Rio San Pedro, Puerto Real, 11510 Cádiz, Spain*

[3]*University Service Centre for Transmission Electron Microscopy, TU Wien, Stadionallee 2/057-02, 1020 Vienna, Austria*

*guenther.rupprechter@tuwien.ac.at

Supporting Note 1: Characterization of as-prepared catalysts

Scanning electron microscopy (SEM)

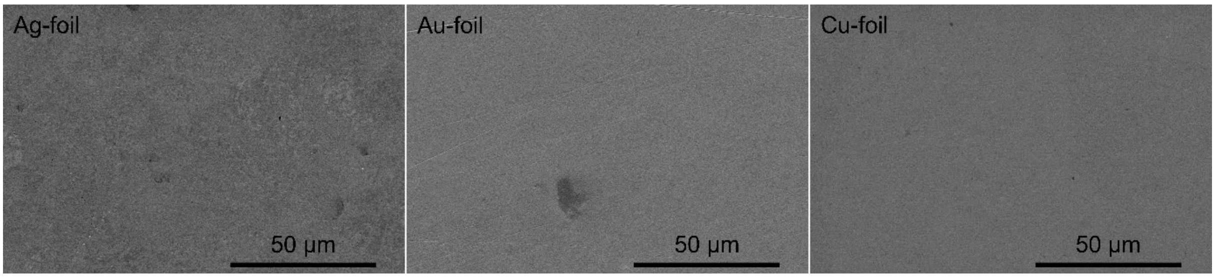

Figure S1. SEM images of the polished Ag-, Au- and Cu-foil.

Energy dispersive X-ray spectroscopy (EDX)

The EDX derived atomic composition of the pristine metal foils is shown in Table S1. Small amounts of Fe, Ni and Al were detected, but disregarded as they stem from the pole piece, the sample holder or other parts of the microscope. No other elements were detected. The foils were transferred through air, explaining the carbon signal.

Table S1. EDX-derived atomic composition of the metal foils.

**Method**	**EDX**		
**Sample**	M /at.%	C /at.%	O /at.%
**Ag-foil**	69.2	27.0	3.8
**Au-foil**	46.9	45.9	7.2
**Cu-foil**	85.8	12.9	1.3

X-ray photoelectron spectroscopy (XPS): Auger regions

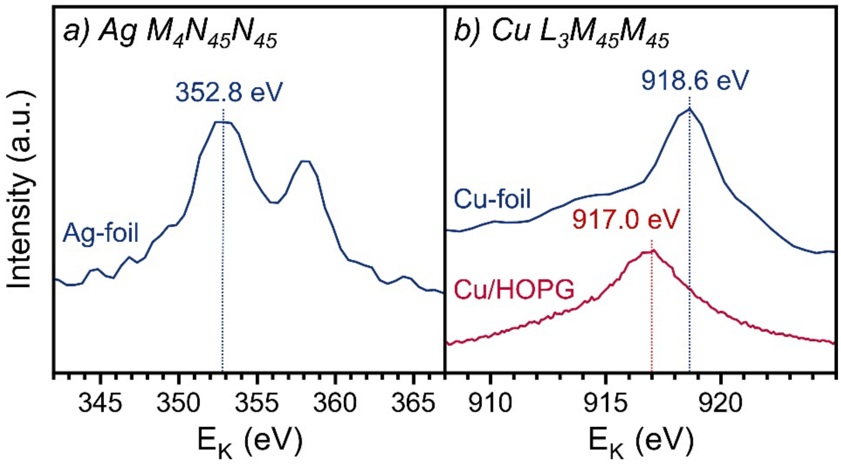

Figure S2. (a) XPS Ag $M_4N_{45}N_{45}$ Auger region of Ag-foil; Ag/HOPG not shown, as the intensity was too low. (b) Cu $L_3M_{45}M_{45}$ Auger region of Cu-foil and Cu/HOPG.

X-ray photoelectron spectroscopy (XPS): O 1s species

Small amounts of residual oxygen were detected for both the NP/HOPG samples as well as the metal foils. As XPS BEs, Auger parameters and LEIS spectra (metal foils only) indicate the absence of metal oxides in all samples except for Cu/HOPG, the origin of the residual O 1s signal should be briefly discussed.

Metal foils: The metal foils were cleaned in UHV by repeated cycles of oxidation, Ar^+ sputtering, reduction and annealing between each step, effectively lowering the carbon and oxygen contamination at the surface. Still, small amounts of oxygen persisted and remained constant in quantity as measured by XPS, even upon prolonged sputtering or reduction. As only minor amounts of oxygen were detected at the surface by LEIS, it is likely that some oxygen was implanted into the subsurface by collisional mixing during Ar^+ sputtering. Additionally, some of the measured oxygen might stem from the steel clips used to fix the metal foils to the sample holder (SH). Reference spectra of the steel foil used for the clips showed a high intensity of the O 1s peak. Although for the measurements of the Ag, Au, and Cu foils, little to no Fe intensity was detected in the survey spectra, some contribution of oxygen from the clips might still be present in the O 1s detail spectra.

NP/HOPG: Between synthesis (by spark ablation) and characterization by XPS in UHV, the NP/HOPG samples were transported through air. Accordingly, the oxygen detected for these samples may originate from adsorbed oxygen species. Additionally, $C_xO_y(H_z)$ species may have formed over time.

Supporting Note 2: Determination of the exposed metal surface atoms (for TOF calculations)

<u>Calculation of the surface atoms of the metal foils</u>

For the number of surface atoms of the metal foils, the atomic density of the (100), (110) and (111) surface terminations was calculated. Figure S3 shows the surface areas A (gray) and the number of atoms N (dark red) of the repeating units of the different planes.

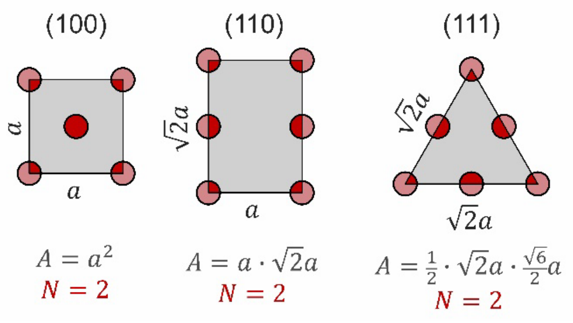

Figure S3. Surface area A (shown in gray) and number of atoms N (shown in dark red) of the repeating units of the (100), (110) and (111) planes. a denotes the lattice parameter.

The surface atomic densities equal N/A. For all orientations, the EBSD-derived area fractions were multiplied with the atomic densities of the closest matching low index surface. The sum of the three values equals the average surface atomic density $\tilde{N}_{M,S}$. The resulting values are shown in Table S2.

Table S2. Distribution of the surface domain orientations from EBSD, lattice parameter a, average surface atomic densities $\tilde{N}_{M,S}$ and the number of exposed surface atoms $N_{M,S}$ of the metal foils and of HOPG.

**Method**	**EBSD**					
**Sample**	(100) /%	(110) /%	(111) /%	a /Å [1]	$\tilde{N}_{M,S}$ /10^{14} cm^{-2}	$N_{M,S}$ /10^{14}
**Ag-foil**	7.5	49.8	42.6	4.09	11.0	4.9
**Au-foil**	14.4	36.7	48.6	4.08	11.6	5.1
**Cu-foil**	99.5	0.2	0.3	3.62	15.3	6.7
		(0001) /%				
**HOPG**[a]		~100		2.46	38.2	16.8

[a] Surface atomic density and number relates to carbon atoms. Surface domain orientation not determined from EBSD, but is along (0001) with low mosaic spread of 0.4° - 0.7°.

Calculation of the metal surface atoms of the HOPG-supported catalysts

While (S)TEM analysis can accurately determine the mean particle size, the total number of NPs in a specific catalyst area is more difficult to obtain (despite about 300 NPs being imaged per catalyst). Thus, a combined (S)TEM/XPS analysis was applied.

First, the number of surface atoms per NP was determined. To simplify, the cuboctahedral NPs were modeled as spheres with a diameter d equal to the mean particle size measured by (S)TEM. From this, one can first calculate the volume V_M of one NP. From V_M and the bulk atomic density of the metal, the number of metal atoms per NP is determined. Multiplying by the dispersion derived from (S)TEM yields the number of surface atoms per NP. Thus, the only information missing is the total number of metal nanoparticles.

This can be expressed by dividing the total surface area of HOPG covered by metal by the support surface area that is covered by one NP: $A_{M,tot}/A_M$. A_M equals the cross-sectional area A_M ($\pi d^2/4$) of one NP. With the known HOPG surface area (A_C, 0.44 cm², 7 x 7 mm², ~10 % covered by steel clips), $A_{M,tot}$ can be calculated by figuring out the ratio $A_{M,tot}/A_C$.

Based on XPS, the intensity (peak area) ratio $I_{M,tot}/I_C$ of the metal and the carbon are known. Now one needs to find a suitable model to figure out the relationship between I_M/I_C and $A_{M,tot}/A_C$. Due to the low metal loadings, the attenuation of I_C by the metal is negligible. I_M is clearly not affected by HOPG as the NPs are on its surface. It is therefore reasonable to model the XPS measurement, as if the two components were measured side by side. The height (thickness) of the HOPG (H_C, 1 mm) is far larger than the inelastic mean free path (IMFP or λ, calculated using QUASES-IMFP-TPP2M Ver.3.0[2,3]) of C 1s electrons traveling through the carbon (λ_{C,E_C}, 33 Å). Therefore, the C 1s intensity can be modeled by a semi-infinite specimen model[4] of a pure HOPG sample. In contrast, the sizes of the nanoparticles (25-40 Å) are on the order of the metal IMFP (λ_{M,E_M}, 10-16 Å). This can be addressed by using a model for films of finite thickness.[4] However, one has to consider the change of the metal nanoparticle height H_C from the center ($r = 0$, $H_C = d$) to the edge of the sphere ($r = d/2$, $H_C = 0$).

Accordingly, the equivalent height $H_{M,Eq}$ of a thin film of equal area A_M was calculated that would lead to the same metal intensity as one spherical particle. This is done by integrating the intensity contribution over the cross-section of the particle ($I_M(H,dA)$, $H(r)=2((d/2)^2-r^2)^{0.5}$, $dA(r)=2\pi r dr$).

Using the equations shown in Figure S4, one calculates the total surface area of HOPG covered by metal $A_{M,tot}$ from $I_{M,tot}/I_C$ and from that one gets the number of metal particles as $A_{M,tot}/A_M$. Multiplying by the earlier derived number of surface atoms per particle finally yields the total number of metal surface atoms in a specific catalyst (Table S3).

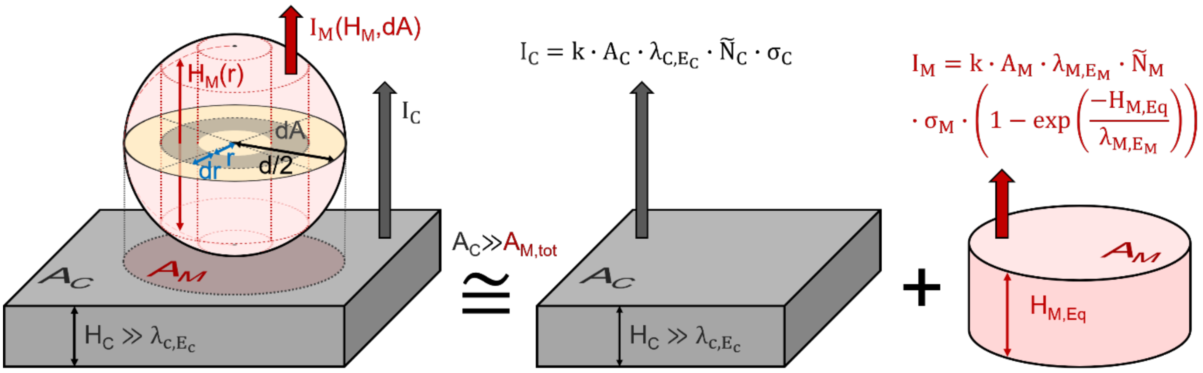

Figure S4. Illustration of the model used to calculate the total number of metal surface atoms of the NP/HOPG samples. The XPS intensity ratio I_M/I_C is roughly the same between the real case in which the approximately round nanoparticles sit on top of HOPG (left) and the model where the HOPG and a metal film of finite height are placed side by side (right). The XPS intensities of the two elements are derived by a constant factor k, as well as from the component specific surface areas A, IMFPs λ, atomic densities $\widetilde{N}$, cross sections σ, and (for the metal) the height (thickness) H.

Table S3. (S)TEM average particle size and dispersion D, ratio of metal covered surface area $A_{M,tot}/A_C$, density of metal nanoparticles N_{NP}, and the number of exposed metal surface atoms $N_{M,S}$ in the HOPG-supported catalysts.

**Method**	**(S)TEM**				
**Sample**	Ø size /nm	D /%	$A_{M,tot}/A_C$ /%	N_{NP} /10^{11} cm^{-2}	$N_{M,S}$ /10^{14}
**Ag/HOPG**	2.8	32	2.6	4.2	0.4
**Au/HOPG**	2.5	37	2.5	5.0	0.4
**Cu/HOPG**	3.9	30	8.3	7.0	2.4

Supporting Note 3: Kinetics

Catalyst deactivation was observed to varying degrees in all catalysts, which poses a challenge, as it can affect the observed reaction orders. We addressed this in a way that allows to compensate for these effects, while still being able to quantify the degree of deactivation of the different catalysts. Deactivation over time leads to lower observed reaction orders when increasing the ethylene (or decreasing the hydrogen) partial pressure and higher observed reaction orders when decreasing the ethylene (or increasing the hydrogen) partial pressure. Therefore, reaction orders were measured consecutively in both directions (increasing and decreasing reactant partial pressures; see Figure 5a,b, Table 2 and Figure S6).

Both RO-E and RO-H were carried out at 50 °C. As can be seen in the Iso-50 run (at 50 °C) in Figure 5d and Figure S5, deactivation was most pronounced in the first 120 min time on stream, after which conversions were quite stable. Similarly, in the RO runs, after ~120 min conversion was rather stable within the 40 min steps at constant feed (Figure S6). Accordingly, one may focus on the values measured in the second half of the run, which are less affected by deactivation.

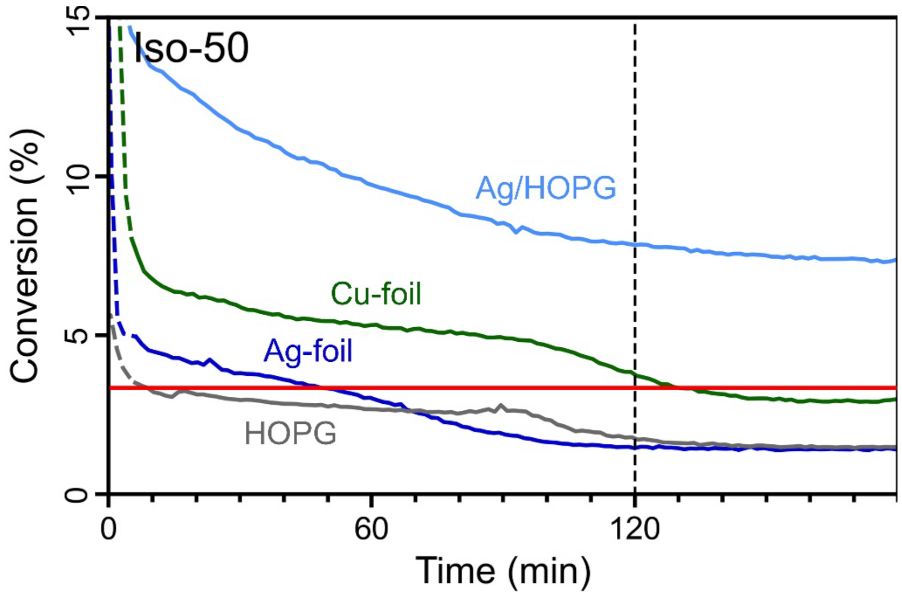

Figure S5. Zoomed in section from Iso-50 run presented in Figure 5d (c.f. Cu/HOPG in Figure 5d). After ~120 min time on stream, conversion is quite stable.

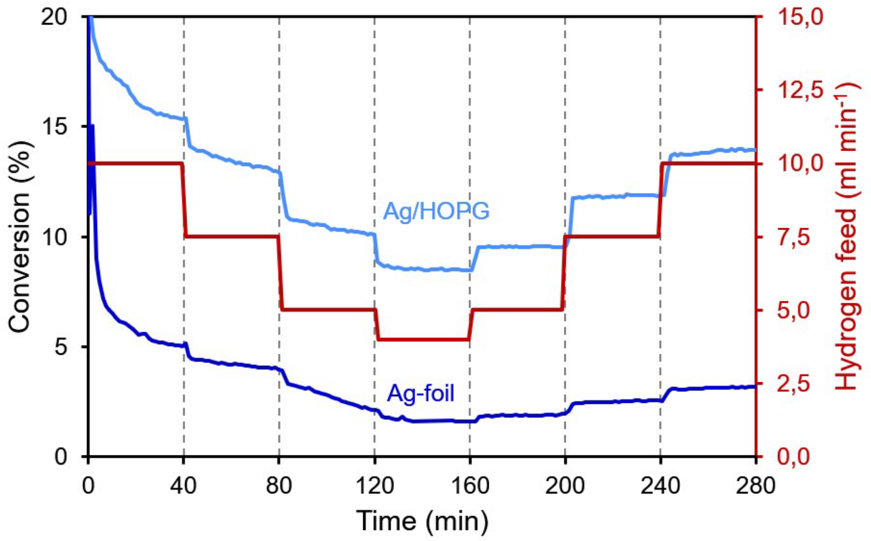

Figure S6. RO-H run for Ag-foil and Ag-HOPG. The hydrogen feed rate was adjusted in stepwise fashion every 40 min. After ~120 min time on stream, conversion is quite stable within the 40 min steps at constant feed.

Figure S7 provides the TOFs for the data shown in Figure 5, in order to better compare the reaction rates of the different catalysts, taking the varying number of surface atoms into account. Au/HOPG and Au-foil were excluded as they showed no significant activity (beyond the blind activity of pure HOPG) and therefore no meaningful normalization based on the number of active sites can be performed as there is no clear active site. For HOPG, normalization was based on the number of carbon atoms (Table S2). Blind activity has not been subtracted (for corrected TOFs, see Figure 6).

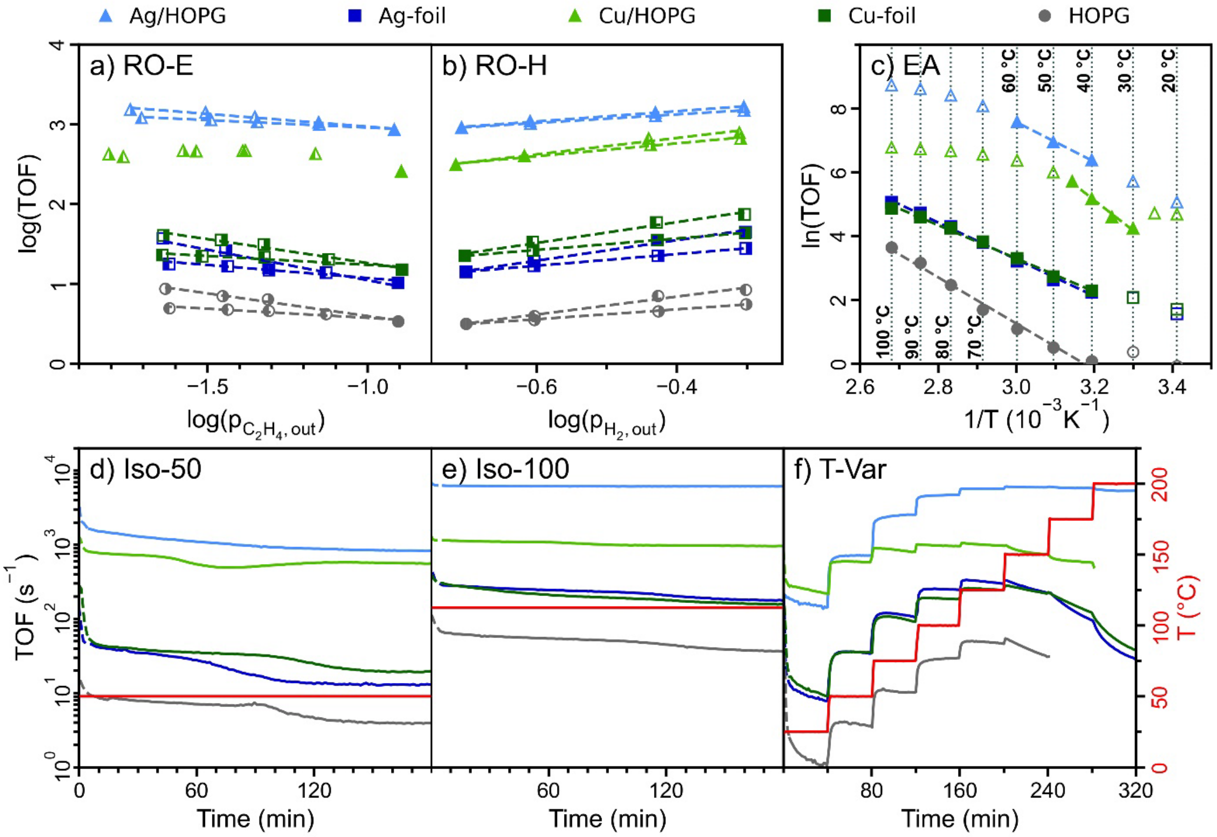

Figure S7. Kinetic data from Figure 5, but using TOFs to compare the different catalysts. The reaction orders of (a) ethylene and (b) hydrogen, (c) the activation energies, the isothermal catalytic performance stability at (d) 50 °C and (e) 100 °C, as well as (f) the overall activity in a broad temperature regime.

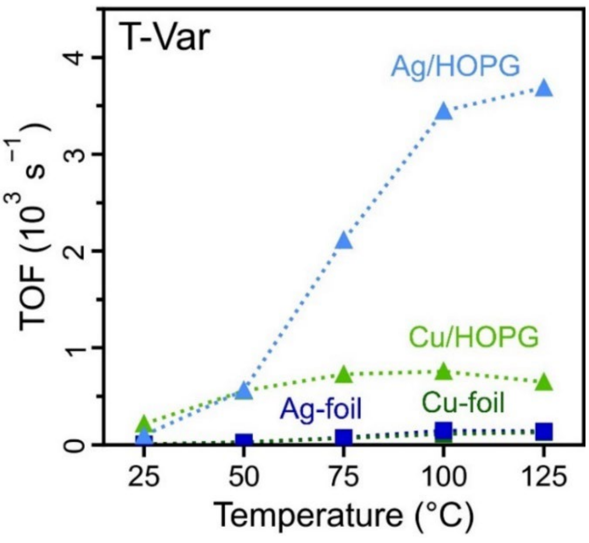

Figure S8. TOFs calculated for the Ag and Cu model catalysts from T-Var experiments. The blind activity of HOPG was subtracted.

Supporting Note 4: Hydrogen-deuterium exchange reaction

The samples were pretreated in the same way as in the ethylene hydrogenation experiments (oxidative and reductive pretreatment) after which the temperature was reduced to 75 °C under a flow of 3 ml/min Ar and 1 ml/min H_2. As the temperature was reached, the gas flow was directed to bypass the reaction cell (pure Ar and H_2 atmosphere inside), maintaining the flow of H_2 and Ar but adding 1 ml/min D_2. Once the gas composition was in equilibrium as measured by the MS, the bypass was switched, redirecting the H_2-D_2 (and Ar) gas mixture into the reaction cell, therefore starting the reaction at 75 °C (Figure S9). Then, after 40 min the temperature was increased from 75 °C to 100 °C and at 80 min to 125 °C. At 120 min, the bypass was opened again, to allow for a reference measurement of the gas intensities without H-D formation. The H_2-D_2 exchange reaction was carried out for Ag/HOPG and Ag-foil with Figure S9 showing the intensities of H_2 and D_2 for the Ag/HOPG run, while comparing the amounts of formed H-D for both Ag catalysts.

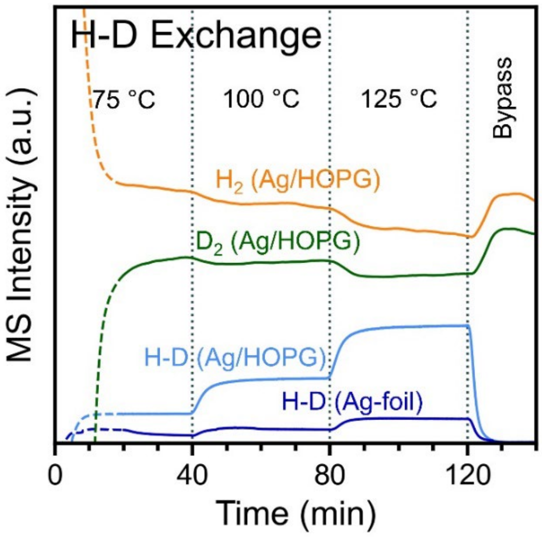

Figure S9. Hydrogen-deuterium exchange reaction at atmospheric pressure with a ratio of 3:1:1 of Ar:H_2:D_2. The mass spectrometer intensity (normalized ion currents) of H_2 (2 u, orange), D_2 (4 u, green) and H-D (3 u, light blue) is shown for the exchange reaction on Ag/HOPG and compared with the H-D intensity of the same exchange reaction on Ag-foil (3 u, dark blue). All intensities were normalized by the intensity of Ar (40 u, not shown), to compensate for possible pressure inconsistencies. In the beginning, some time is needed until the gas composition is in equilibrium (dashed lines).

Supporting Note 5: DFT calculations of reactants and intermediates

DFT modeling of ethylene adsorption on low index Ag surfaces revealed only moderately favorable adsorption with E_{ads} (Ethylene) ~ -0.4 eV, which at a bare Ag nanoparticle (NP) was somewhat stronger (~ -0.7 eV). At a graphene-supported Ag NP, ethylene adsorbs in a range from -1.0 to -1.3 eV, near the interface favored by ~ -0.55 eV when compared to adsorption at the bare particle (see Table S4 for values and Figure S10 for the location of the adsorption sites).

Considering the reaction energy of C_2H_4* + H* → C_2H_5* + *(adsorption site), one notes that this reaction is always exothermic at low index surfaces, ranging from -0.3 to -0.8 eV. At unsupported Ag NPs, the reaction energy spans from 0.0 to -0.6 eV. For graphene-supported Ag_{37} it is mostly endothermic/thermoneutral, ranging from +0.5 to -0.2 eV. Thus, the first hydrogenation step is favorable at all sites of the single crystal surfaces and the bare NP, but only at specific sites of carbon-supported Ag_{37}. This may lead to a "less C_2H_5* crowded" situation at the supported particle (note that hydrocarbon species may block hydrogen adsorption).[5,6]

Ethyl binds rather strong on all surface types, but weakest at the low index surfaces with $E_{ads}(C_2H_5)$ from -1.1 to -1.2 eV. It binds stronger at a bare NP (~ -1.5 eV), and again strongest at the graphene-supported Ag NP with -1.7 to -2.1 eV. This translates to a favorable second hydrogenation energy for all surfaces, C_2H_5* + H* → C_2H_6* + *, C_2H_6* in Table S4. The reaction energy ranges from -1.4 to -1.9 eV at single crystal surfaces, from -0.8 to -1.5 eV at a bare Ag NP, and from -0.3 to -1.0 eV at graphene-supported Ag NPs. Consequently, when ethyl has been formed, the second hydrogenation step is a downhill process at all sites.

The product ethane binds reasonably strong on all surfaces, -0.2 to -0.3 eV at low index Ag surfaces, -0.3 to -0.4 eV at Ag NPs, and -0.8 to -1.0 eV at graphene-supported Ag particle. Hence, desorption of the product is most facile without carbon support, but the reactants bind sufficiently strong to displace the product. To check if a similar trend applies to an alternative metal, H_2 and H adsorption was calculated also at a Cu_{37} particle for the most promising sites. Although the effect of graphene is weaker, the adsorption energy becomes more favorable by 0.1 eV due to the presence of the carbon support (Table S4).

An alternative rationalization of the effect of carbon might be a possible incorporation of sub-surface carbon atoms. To study this possibility, a model of Ag_{37} was used where a carbon was placed under the top layer of the (100) facet and the values for H_2 and H adsorption were calculated (Figure S11). As expected, the sub-surface carbon moiety results in a weaker binding of both H_2 and H by 0.1 to 0.6 eV (Table S5). For the most preferred site of H at the interface of the (100) facet to the carbon support, a decrease in binding of 0.35 eV is evident. Similarly, at the interface of the edge, a reduction in binding by 0.50 eV is noticed for H_2 (Figure S11).

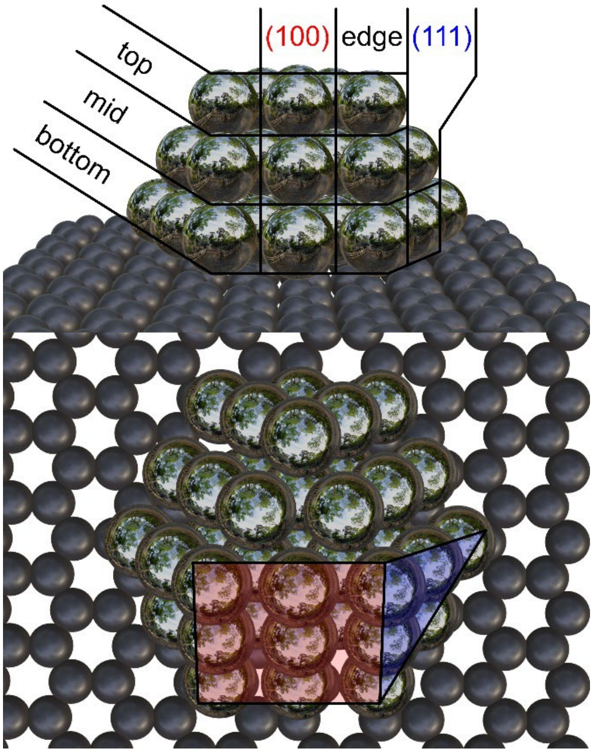

Figure S10. The graphene-supported Ag_{37} model with adsorption sites indicated.

Table S4. Adsorption energies per adsorbate and reaction energies leading to the designated final product, both for Ag single crystals and unsupported and supported Ag_{37} and Cu_{37} particles. The most stable site is marked in bold face. L denotes the location of the adsorption site in the top (t), mid (m), or bottom (b) layer. S labels the location of the site, either (100) facet, edge, (111) facet, cf. Figure S10. Δ support indicates the adsorption energy difference between the most stable site of a graphene-supported and unsupported Ag particle. An asterisk indicates an adsorbed species.

			Adsorption Energy (eV)					Reaction Energy (eV)		
System	L	S	H	H_2	C_2H_4	C_2H_5	C_2H_6	H*	C_2H_5*	C_2H_6*
Ag(111)		fcc	**-2.26**	-0.07	-0.40	-1.14	-0.28	**0.04**	-0.29	-1.39
		Bridge	-2.14	-0.07	-0.45	**-1.17**	**-0.29**	0.17	-0.40	-1.49
		on top	-1.72	-0.07	**-0.46**	**-1.18**	**-0.29**	0.58	**-0.81**	**-1.89**
Ag(100)		hollow	**-2.16**	-0.07	-0.37	-1.15	**-0.29**	**0.14**	-0.42	-1.49
		bridge	**-2.14**	-0.07	-0.49	**-1.17**	-0.17	0.17	-0.36	-1.38
		on top	-1.71	-0.07	**-0.50**	-1.09	-0.18	0.59	**-0.69**	**-1.88**
Ag NP	t	(100)	**-2.49**[a]	-0.23	-0.62	-1.32	-0.37	-0.10	-0.02	-1.07
	m	(100)	-2.35	-0.24	-0.54	-1.26	**-0.41**	0.04	-0.18	-1.31
	b	(100)	**-2.64**	-0.25	-0.66	**-1.46**	-0.37	**-0.25**	0.03	-0.77
	t	edge	-1.97	**-0.26**	**-0.75**[a]	-1.36	-0.35	0.43	-0.46	**-1.53**
	m	edge	-2.44[a]	-0.24	-0.63	-1.29	-0.35	-0.05	-0.04	-1.13
	b	edge	-1.94	-0.25	**-0.74**	**-1.48**	-0.35	0.45	**-0.61**	-1.43
	b	(111)	-2.53	-0.23	-0.55[a]	-1.35	-0.32	-0.14	-0.09	-0.96
Ag NP on graphene	t	(100)	-2.92	-0.66	-1.04	-1.80	-0.76	-0.32	0.35	-0.56
	m	(100)	-2.80	-0.69	-0.99	-1.71	-0.87	-0.18	0.27	-0.88
	b	(100)	**-3.12**	-0.73	-1.24	-2.04	**-0.99**	**-0.49**	0.51	-0.33
	t	edge	-2.48	-0.70	-1.21	-1.86	-0.80	0.14	0.01	**-0.97**
	m	edge	-2.91	-0.71	-1.09	-1.76	-0.83	-0.29	0.43	-0.67
	b	edge	-2.42	**-0.75**	**-1.30**	**-2.08**	-0.96	0.22	**-0.17**	**-0.97**
	b	(111)	-3.02	-0.74	-1.15	-1.95	-0.93	-0.38	0.40	-0.48
Δ support			-0.48	-0.49	-0.55	-0.60	-0.58	-0.24	0.44	0.56
Cu NP	b	(100)	**-2.69**							
	b	edge		**-0.20**						
Cu NP on graphene	b	(100)	**-2.79**							
	b	edge		**-0.29**						

[a] Initially, this configuration led to a deformed particle. Therefore, the “bottom” layer of the NP was fixed to enforce a consistent shape for all adsorption complexes.

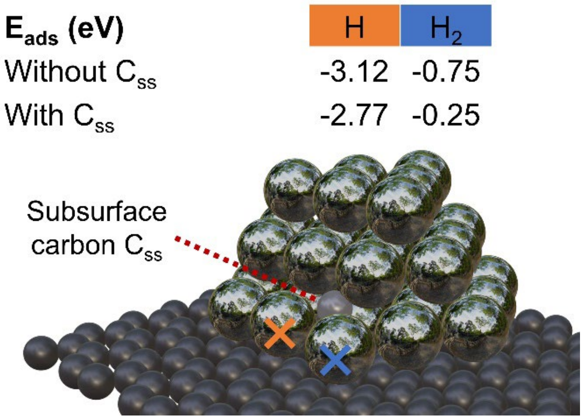

Figure S11. The graphene-supported Ag_{37} nanoparticle model with subsurface carbon (C_{ss}). The adsorption energies of atomic and molecular hydrogen are higher without the presence of C_{ss}.

Table S5. Adsorption energy of H and H_2 moieties at graphene-supported Ag_{37} particles with and without a subsurface carbon (C_{ss}).

			Without C_{ss}		With C_{ss}		Δ C_{ss}	
System	L	S	H	H_2	H	H_2	H	H_2
Ag NP on graphene	t	(100)	-2.92	-0.66	-2.31	-0.05	0.61	0.61
	m	(100)	-2.80	-0.69	-2.27	-0.08	0.53	0.61
	b	(100)	**-3.12**	-0.73	**-2.77**	-0.15	**0.35**	0.58
	t	edge	-2.48	-0.70	-2.33	-0.09	0.15	0.61
	m	edge	-2.91	-0.71	-2.37	-0.13	0.54	0.58
	b	edge	-2.42	**-0.75**	-2.29	**-0.25**	0.13	**0.50**
	b	(111)	-3.02	-0.74	-2.60	-0.15	0.42	0.59

Supporting Note 6: Post reaction morphology analysis of the metal nanoparticles

To examine whether the metal NPs had changed under reaction conditions, (S)TEM analysis was repeated after the samples were pretreated (oxidative and reductive) and subjected to reaction conditions at 100°C for several hours (Figure S12). As shown in Table S6, the mean particle size slightly increased for Ag-NPs, more for Au-NPs, and somewhat decreased for Cu-NPs.

An increase in particle size will lead to a decrease in dispersion. Accordingly, assuming cuboctahedra particle shape, the actual TOFs of the Ag/HOPG catalyst may even be ~30 % higher, as the TOF calculations were based on (S)TEM and XPS data of as-prepared samples. For the rather inactive Au/HOPG a correction is irrelevant and for Cu/HOPG the size change is rather small. Therefore, all activity data reported herein were based on as-prepared catalysts.

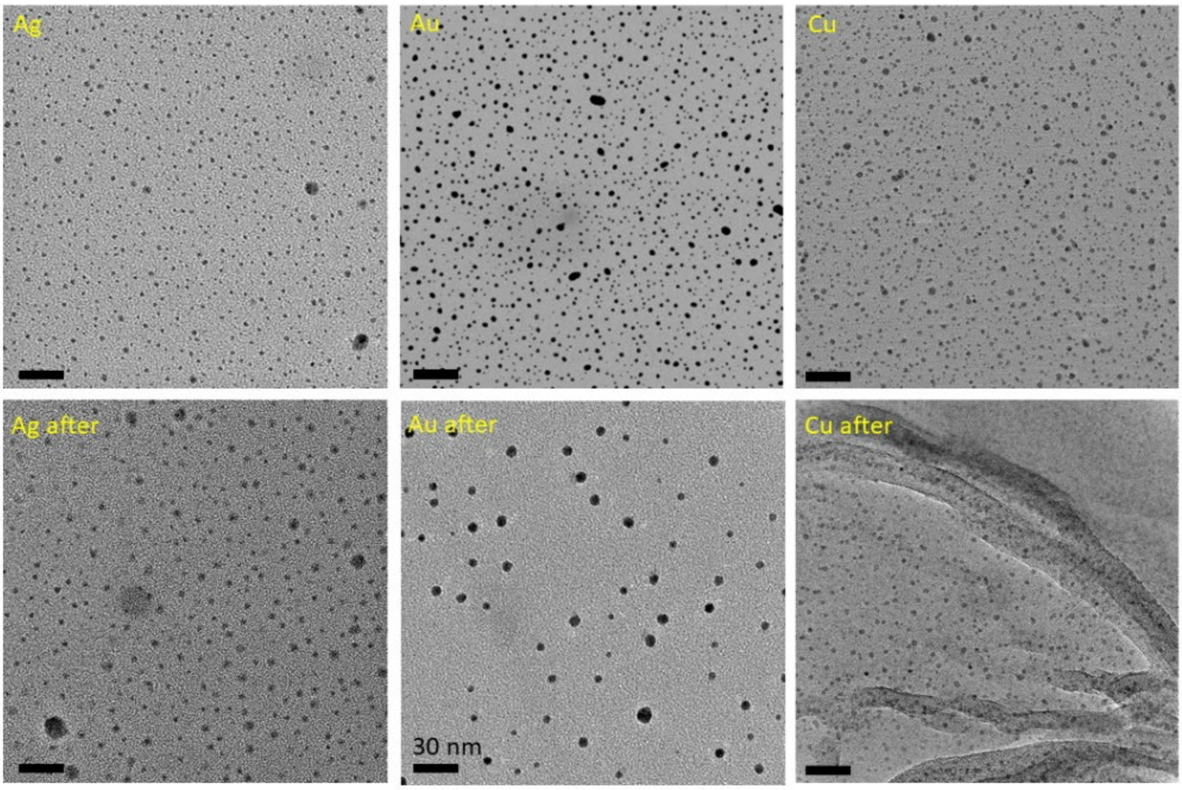

Figure S12. Low magnification TEM images of the supported metal NPs before and after exposure to reaction conditions. The scale bar is 30 nm in all cases.

Table S6. Comparison of particle sizes and dispersions measured by (S)TEM after synthesis and after pretreatment and exposure to reaction conditions.

TEM	Ø size /nm		D /%	
**Sample**	as-synthesized	post reaction	as-synthesized	post reaction
**Ag/HOPG**	2.8	3.6	32	23
**Au/HOPG**	2.5	5.8	37	20
**Cu/HOPG**	3.9	3.1	30	37

Figure S13 shows high-resolution lattice images of the supported NPs, both as-prepared and after reaction. For the most active Ag NPs, rounded NPs with fcc structure were observed, so that cuboctahedral particle shape is a fair assumption. The Au NPs were mostly multiply twinned particles (MTPs) of decahedral shape and they maintained the rounded/stepped surface and internal defects (twin boundaries) during reaction. The Cu NPs were similar to the Ag ones, with rounded shapes. Altogether, apart from size changes, electron microscopy analysis did not indicate any major changes in particle shape, surface or defect structure.

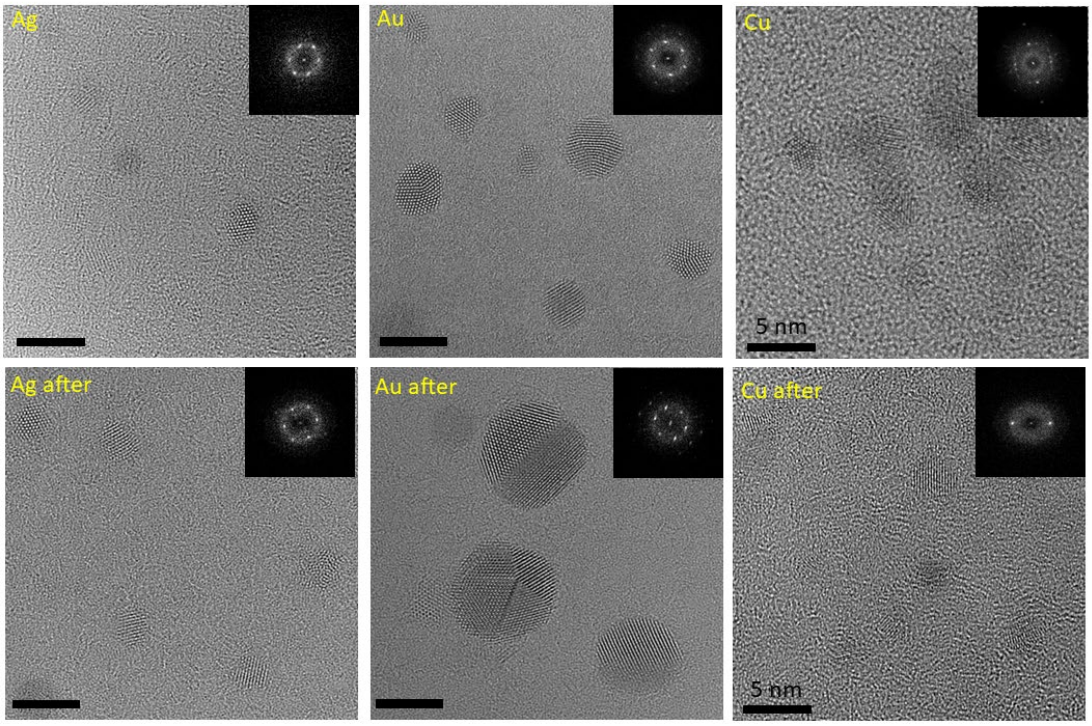

Figure S13. High resolution TEM images of the supported metal NPs before and after exposure to reaction conditions. The scale bar is 5 nm in all cases.

Supporting Note 7: Deactivation studies

To examine the observed deactivation in more detail, all samples were once more pretreated and exposed to reaction conditions at 200 °C for 1 h. Subsequently, the samples were transferred from the microreactor to the UHV chamber for XPS (Table S7). For all HOPG-based samples, the "nominal" metal concentration decreased, the carbon concentration hardly changed, but the oxygen signal increased. As the reaction is carried out under reducing conditions, this suggests metal oxidation during sample transfer in air.

The deactivation results were more pronounced for the polycrystalline metal foils. The "nominal" metal concentrations decreased 2.5-14 times as the carbon signals increased strongly. The O 1s was only slightly higher, as the foils were less easily oxidized during transfer than NPs. Altogether, the combined (S)TEM and XPS analysis suggests carbon coking of metal surfaces as the main reason of deactivation.

Table S7. Comparison of XPS data before and after deliberate deactivation at 200 °C.

**XPS**	as-synthesized			post deactivation		
**Sample**	c_M /at.%	c_C /at.%	c_O /at.%	c_M /at.%	c_C /at.%	c_O /at.%
**Ag/HOPG**	0.4	95.6	4.0	0.3	93.1	6.7
**Au/HOPG**	0.4	99.0	0.6	0.3	97.4	2.3
**Cu/HOPG**	1.5	91.7	6.8	0.9	92.8	6.3
**HOPG**	0	98.8	1.2	0	95.6	4.4
**Ag-foil**	84.6	1.7	13.7	5.9	80.2	13.9
**Au-foil**	80.1	14.9	5.1	30.0	50.4	19.5
**Cu-foil**	59.4	18.1	22.6	17.5	56.2	26.4

References

(1) Ashcroft, N. W.; Mermin, N. D. *Solid State Physics, College edn*; 1976.
(2) Tanuma, S.; Powell, C. J.; Penn, D. R. Calculations of electron inelastic mean free paths. V. Data for 14 organic compounds over the 50–2000 eV range. *Surf. Interface Anal.* **1994**, *21* (3), 165-176. DOI: 10.1002/sia.740210302.
(3) Tougaard, S. M. QUASES-Inelastic electron mean free path calculator (by TPP2M formula). **2021**.
(4) Fadley, C. S. Basic concepts of X-ray photoelectron spectroscopy. In *Electron spectroscopy: theory, techniques and applications*, Vol. 2; 1978; pp 1-156.
(5) Morkel, M.; Rupprechter, G.; Freund, H.-J. Finite size effects on supported Pd nanoparticles: Interaction of hydrogen with CO and C_2H_4. *Surface Science* **2005**, *588* (1-3), L209-L219. DOI: 10.1016/j.susc.2005.05.037.
(6) Rupprechter, G.; Morkel, M.; Freund, H.-J.; Hirschl, R. Sum frequency generation and density functional studies of CO–H interaction and hydrogen bulk dissolution on Pd(111). *Surface Science* **2004**, *554* (1), 43-59. DOI: 10.1016/j.susc.2004.02.008.
